# Supplementary material for: Learning lessons from over-crediting to ensure additionality in forest carbon credits
Source: Nat Commun. 2026 Apr 11;17:3944. doi: 10.1038/s41467-026-71552-3 (PMC13133336; doi:10.1038/s41467-026-71552-3)
Supplement: Supplementary file 1 — Supplementary Information [file 41467_2026_71552_MOESM1_ESM.pdf]

Supplementary Information for

Learning lessons from over-crediting to ensure additionality in forest carbon credits

Tom Swinfield, Abigail E. Williams, David Coomes, Michael Dales, Patrick Ferris, Alejandro Guizar-Coutiño, James Hartup, Jody Holland, Sadiq Jaffer, Julia P G Jones, Miranda Oi Ki Lam, Srinivasan Keshav, Anil Madhavapeddy, Eleanor Toye-Scott, Thales A. P. West, Andrew Balmford.

Correspondence to: tws36@cam.ac.uk

This document includes:

Supplementary note 1. Contrasting methods to assess REDD+ impact.
Supplementary table 1. Projects included in each analysis.
Supplementary figure 1. Comparison of quasi-experimental and certified estimates of project performance labelled by project.
Supplementary figure 2. Comparison of quasi-experimental and certified estimates of project performance coloured by country where the project was implemented.
Supplementary figure 3. The remotely sensed deforestation rates in project areas when forest cover is defined as undisturbed or degraded.
Supplementary figure 4. The remotely sensed deforestation rates in project areas when forest cover is defined as undisturbed, degraded or regrowth.
Supplementary figure 5. The remotely sensed deforestation rates in project areas when deforestation is defined as loss of undisturbed or degraded ACC classes.
Supplementary figure 6. The remotely sensed deforestation rates in project areas when deforestation is defined as loss of undisturbed or regrowth ACC classes.
Supplementary figure 7. The location of the project, certified reference and PACT control areas, and differences in exposure to deforestation drivers for project 944**.**
Supplementary figure 8. The location of the project, certified reference and PACT control areas, and differences in exposure to deforestation drivers for project 958**.**
Supplementary figure 9. The location of the project, certified reference and PACT control areas, and differences in exposure to deforestation drivers for project 1047**.**
Supplementary figure 10. The location of the project, certified reference and PACT control areas, and differences in exposure to deforestation drivers for project 1094**.**
Supplementary figure 11. The location of the project, certified reference and PACT control areas, and differences in exposure to deforestation drivers for project 1112**.**
Supplementary figure 12. The location of the project, certified reference and PACT control areas, and differences in exposure to deforestation drivers for project 1113**.**
Supplementary figure 13. The location of the project, certified reference and PACT control areas, and differences in exposure to deforestation drivers for project 1115**.**

Supplementary figure 14. The location of the project, certified reference and PACT control areas, and differences in exposure to deforestation drivers for project 1201.
Supplementary figure 15. The location of the project, certified reference and PACT control areas, and differences in exposure to deforestation drivers for project 1329.
Supplementary figure 16. The location of the project, certified reference and PACT control areas, and differences in exposure to deforestation drivers for project 1400.
Supplementary figure 17. The location of the project, certified reference and PACT control areas, and differences in exposure to deforestation drivers for project 1566.
Supplementary figure 18. The location of the project, certified reference and PACT control areas, and differences in exposure to deforestation drivers for project 1571.
Supplementary figure 19. The location of the project, certified reference and PACT control areas, and differences in exposure to deforestation drivers for project 1650.
Supplementary figure 20. The remotely sensed deforestation rates in control areas when deforestation is defined as loss of undisturbed or degraded.
Supplementary figure 21. The remotely sensed deforestation rates in control areas when deforestation is defined as loss of undisturbed or regrowth.
Supplementary figure 22. The remotely sensed deforestation rates in control areas when deforestation is defined as loss of undisturbed, degraded or regrowth.
Supplementary figure 23. The avoided deforestation rates for the 17 projects in the main analysis (solid lines in Fig. 5) are compared with avoided deforestation rates that also include transitions from the regrowth class to the deforested class (dashed lines)
Supplementary note 2. Differences in avoided deforestation estimates produced by alternative certified modelling approaches.
Supplementary table 2. Modelling approaches, available under different certification methods, typically produced higher estimates of avoided deforestation than quasi-experimental methods, and projects used methods which produced improbably high estimates.
Supplementary table 3. Details of the calculation of avoided deforestation using the numbers extracted from the most recent VERRA monitoring reports for each project included in our assessment.
Supplementary table 4. Details of the calculation of avoided deforestation using the numbers produced by PACT for each project included in our assessment.
Supplementary table 5. The CO_2e (t) of each ACC land cover class as measured by GEDI in each project.
Supplementary figure 24. The mean carbon densities of each ACC forest and cover class as from GEDI across 39 projects.
Supplementary table 6. Details of the calculation of certified compound annual deforestation rates.
Supplementary table 7. Comparison of compound annual deforestation rates in PACT counterfactuals, projects and certified reference areas measured using the ACC.
Supplementary references.

Supplementary note 1. Contrasting methods to assess REDD+ impact.

In this section we summarise the overarching process of assessing REDD+ projects with certification and quasi-experimental methods. In both approaches, assessors possessed a degree of flexibility in decision-making that may have affected outcomes. Within certification methods, assessors had flexibility over the selection of the control area, the historic reference period, the remotely sensed forest classification and the *ex ante* model of future deforestation. For quasi-experimental methods, there was flexibility in the selection of pre-project characteristics, rules for determining the domain of untreated units, the remotely sensed forest classification, and the statistical model for selecting control units.

Certification methods

The methods used in the assessments of the first generation of REDD+ projects were taken from a set of available certification methods. While these methods differed in their detail, which is documented by Verra [1] and explained comprehensively in West et al. [2], they follow a broadly similar approach:

1. **Define the project area.** REDD+ projects may have inherited their boundaries from pre-existing protected areas but they must also have conformed to the applicability conditions of certification methods. This means projects must have possessed suitable forest cover for at least the preceding 10 years, as demonstrated using remotely sensed evidence. A project also specified a start date after which the intervention came into effect.
2. **Define the control area.** Control areas (also known as the reference area) were defined using a combination of geo-spatial layers. These control areas were selected so that they had similar characteristics to the project in terms of forest cover, and expected drivers of deforestation. Control areas were determined to be similar to projects if their characteristics fell within the range of values observed within the project (± percentage tolerance outside the range).
3. **Measure historic deforestation in the control area.** Historic deforestation figures were extracted from forest cover classification maps. These maps were typically produced from satellite remote sensing. Classification typically involved training a model to minimise errors when predicting a spatial dataset of forest and non-forest classes, produced from field or high-resolution aerial observations. It is well known that different results can be produced by changing the set of forest / non-forest observations, the satellite imagery or the underlying model. To estimate deforestation rates, measurements were made of the area of forest at the start of the project and at two or more historic time points. The period was often the same as the period used to check the applicability condition under (1), because it required the same assessment of forest cover. Thus, the availability of suitable cloud free satellite imagery determined the historic reference period during which deforestation rates were measured. The mean length of reference periods in our dataset was found to be 13.6 years.
4. **Predict *ex ante* counterfactual deforestation for the project.** REDD+ projects must have been forest for the preceding 10 years. Thus, projects should have no historic deforestation by definition. Therefore, control areas were effectively a space-for-time substitute for the project, assumed to be equivalent in terms of

probability of becoming a REDD+ project and exposure to the drivers of deforestation, but several years ahead in terms of the deforestation experienced. *Ex ante* counterfactual deforestation rates were estimated using one of a variety of possible methods. These spanned averaging historic rates, regressing linear and non-linear changes through time, and modelling spatial deforestation patterns using a risk modelling approach. Spatial risk models were developed over an area that included but was larger than the project (also known as the reference area for location). Deforestation was then allocated across the reference area for location in order of risk probability at the rate measured in (3). This meant that if the project was estimated to be at relatively high risk it would be allocated a larger proportion of the deforestation, and could experience a higher rate than the control area.

5. **Measure *ex post* deforestation in the project area.** A remote sensing classification is produced for the project at a point in time subsequent to the project commencing. The difference between the assessed forest cover at the time of monitoring and the start of the project provides the *ex post* measure of project deforestation.
6. **Calculate avoided deforestation by comparison of the project and counterfactual.** The *ex post* measurement of project deforestation is subtracted from the *ex ante* estimate for the counterfactual scenario for the same point in time to calculate avoided deforestation.
7. **Verify project assessment**. The report produced by the project proponent is submitted to a third-party auditor who verifies that the method hosted by the standard has been appropriately followed. Where methods have not been followed appropriately, assessments require revision.

Note that current jurisdictional methods follow a similar approach, but the project and control areas (for measuring historic deforestation and predicting risks) are both defined as the jurisdiction itself.

Quasi-experimental methods

Different quasi-experimental methods are available to assess the effect of REDD+ interventions on deforestation.These methods differ in their approaches and input data [3–9] are broadly similar in their structure and assumptions:

1. **Define the project area.** A spatial polygon and start date were accessed from an online repository.
2. **Define the control area.** A set of geospatial layers were compiled that encoded pre-project characteristics known to be associated with the selection of projects and deforestation (i.e. likely confounders). These pre-project characteristics could represent point specific features such as elevation or slope, or neighbourhood statistics covering a larger area such as surrounding forest cover. For each project, a sample was taken representing project exposure to these pre-project characteristics. Sampling required a specified unit size and technique: Unit sizes ranged from whole project areas to pixels at the highest geospatial resolution. For smaller units sizes, sampling typically occurred across a grid covering the project polygon. Pre-project samples were usually summarised as the mean and range of the various pre-project characteristics. Next the domain of possible untreated units was determined by filtering to locations that were identical to projects according to categorical

characteristics, for example within the same country and biome, not within another REDD+ project, and less than a specified maximum distance from the project. Across this domain and utilising the pre-project characteristics, a statistical method (Matching or Synthetic Control) was employed to select a set of untreated control units, which collectively form a control area equivalent to the project. Post-hoc tests were used to assess the validity of this control area.

3. **Measure *ex post* deforestation in the project and control areas.** A peer-reviewed forest cover classification was used to measure changes in forest cover in the project and control areas across the same time period. For both project and control areas, the difference between the forest cover at the time of monitoring and the start of the project provides an *ex post* measure of deforestation.
4. **Calculate avoided deforestation by comparison of the project and counterfactual.** The *ex post* measurement of project deforestation is subtracted from the *ex post* measurement of control area deforestation (which functions as an estimated counterfactual scenario) to calculate avoided deforestation. The requirement for REDD+ projects to be defined as 100% forested when they start makes it impossible to apply difference in difference methods as the pre-project deforestation rate should always be zero [8].
5. **Verify project assessment**. A quasi-experiment REDD+ assessment can be verified through third-party reproduction of results using the clearly stated method, publicly available datasets, and open-source code. In addition, peer-review ensures that up-to-date credible methods and data have been used.

Supplementary table 1. Projects included in each analysis. Different subsets of projects were included in each analysis based on the information available. 46 projects analysed by the different statistical analyses that had certified amounts of avoided deforestation were included in Question 1. Question 2 required that project area deforestation rates were included in certified documents, this amounted to 36 projects. Question 3 required that reference areas could be acquired with reasonable accuracy from maps provided in the Project Design Documents; this applied to 17 projects. Question 4 covered the same 17 projects.

Project	Country	Q1 (Fig. 2)	Q2 (Fig. 3)	Q3 (Fig. 4)	Q4 (Fig. 5)
844	Peru	x	x		
856	Colombia	x	x		
875	Brazil	x	x		
904	Cambodia	x			
934	Congo	x	x		
944	Peru	x	x	x	x
958	Peru	x	x	x	x
963	Brazil	x	x		
977	Brazil	x			
981	Brazil	x	x		
985	Peru	x	x		
1047	Madagascar	x	x	x	x
1067	Peru	x	x		
1094	Brazil	x	x	x	x
1112	Brazil	x	x	x	x
1113	Brazil	x	x	x	x
1115	Brazil	x	x	x	x
1118	Brazil		x		
1201	Sierra Leone	x	x	x	x
1202	Zambia	x			
1215	Madagascar	x	x		
1218	Peru	x			
1311	Madagascar	x	x		
1326	Belize	x	x		
1329	Brazil	x	x	x	x
1359	D.R. Congo	x			
1360	Peru	x	x		
1382	Brazil	x	x		
1389	Colombia	x	x		
1390	Colombia	x	x	x	x
1391	Colombia	x	x		
1392	Colombia	x	x	x	x
1395	Colombia	x	x		
1396	Colombia	x	x		
1399	Colombia	x	x	x	x
1400	Colombia	x	x	x	x
1503	Brazil	x	x		
1566	Colombia	x	x	x	x
1571	Brazil	x	x	x	x
1650	Cambodia	x	x	x	x
1686	Brazil	x	x		
1882	Peru	x	x		
1897	Tanzania	x			

1900	Tanzania	x			
2278	Peru	x			
**Total**		**44**	**36**	**17**	**17**

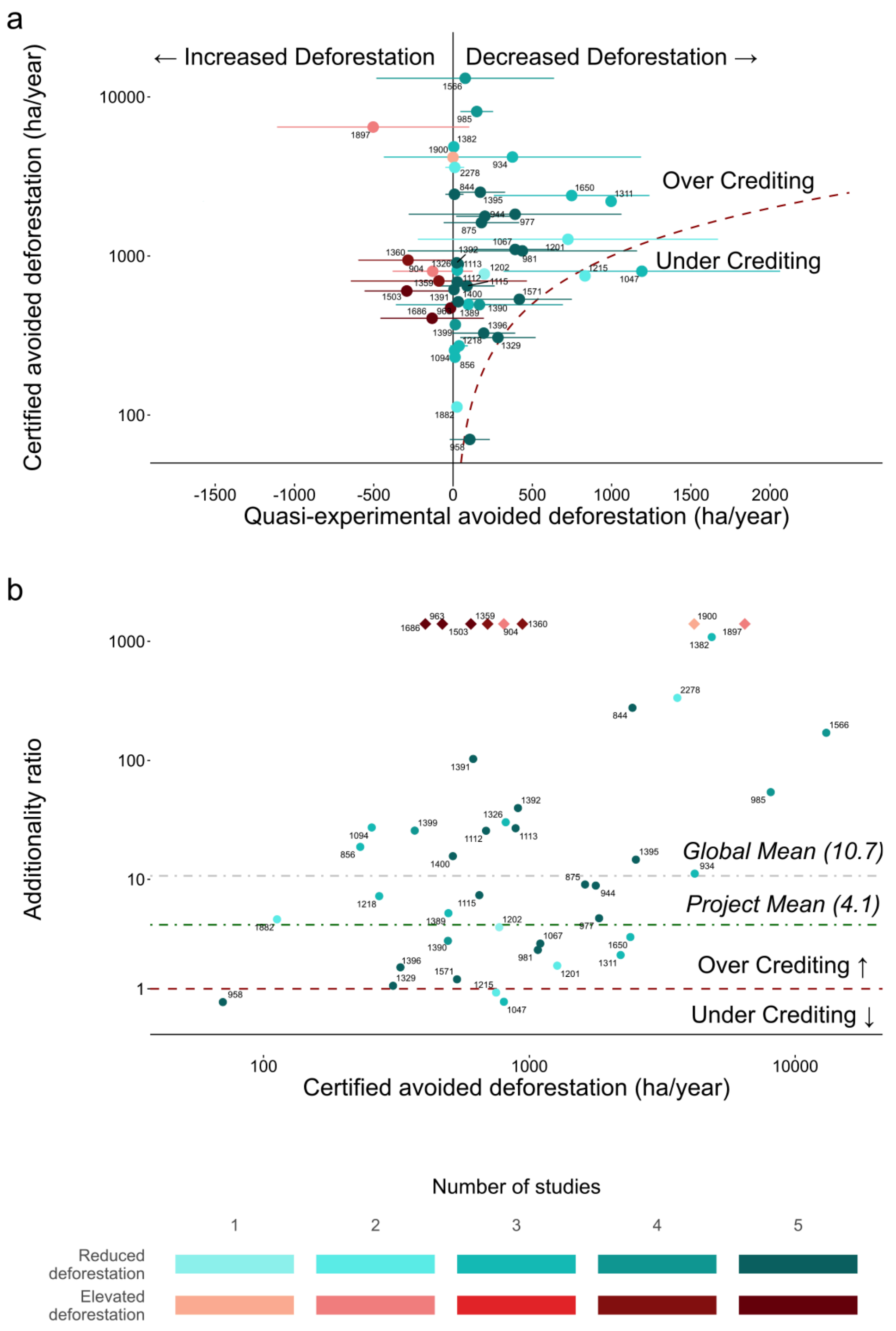

Supplementary figure 1. Comparison of quasi-experimental and certified estimates of project performance labelled by project. To enable interpretation of over-crediting ratios of individual projects, we have recreated figure 1 but with points labelled by the Verra project ID

numbers. (a) 44 REDD+ projects' certified estimates of avoided deforestation against mean estimates from the five quasi-experimental methods, and (b) the over-crediting ratio (the mean quasi-experimental estimate divided by the certified estimate) against certified estimates of avoided deforestation. In both plots certified estimates of avoided deforestation are shown on logarithmic scales. Dashed black lines show where quasi-experimental and certified estimates are equal. In (b) the solid and dashed grey lines show the mean over-crediting ratio weighting projects equally and weighting them by the size of their certified estimates, respectively. Darker colours indicate projects that were assessed by a greater number of quasi-experimental approaches (to a maximum of five because there was no overlap in the projects assessed by the two West et al. studies). Hollow points show projects assessed to have negative additionality by certified assessments. Confidence intervals show 1.96 standard errors.

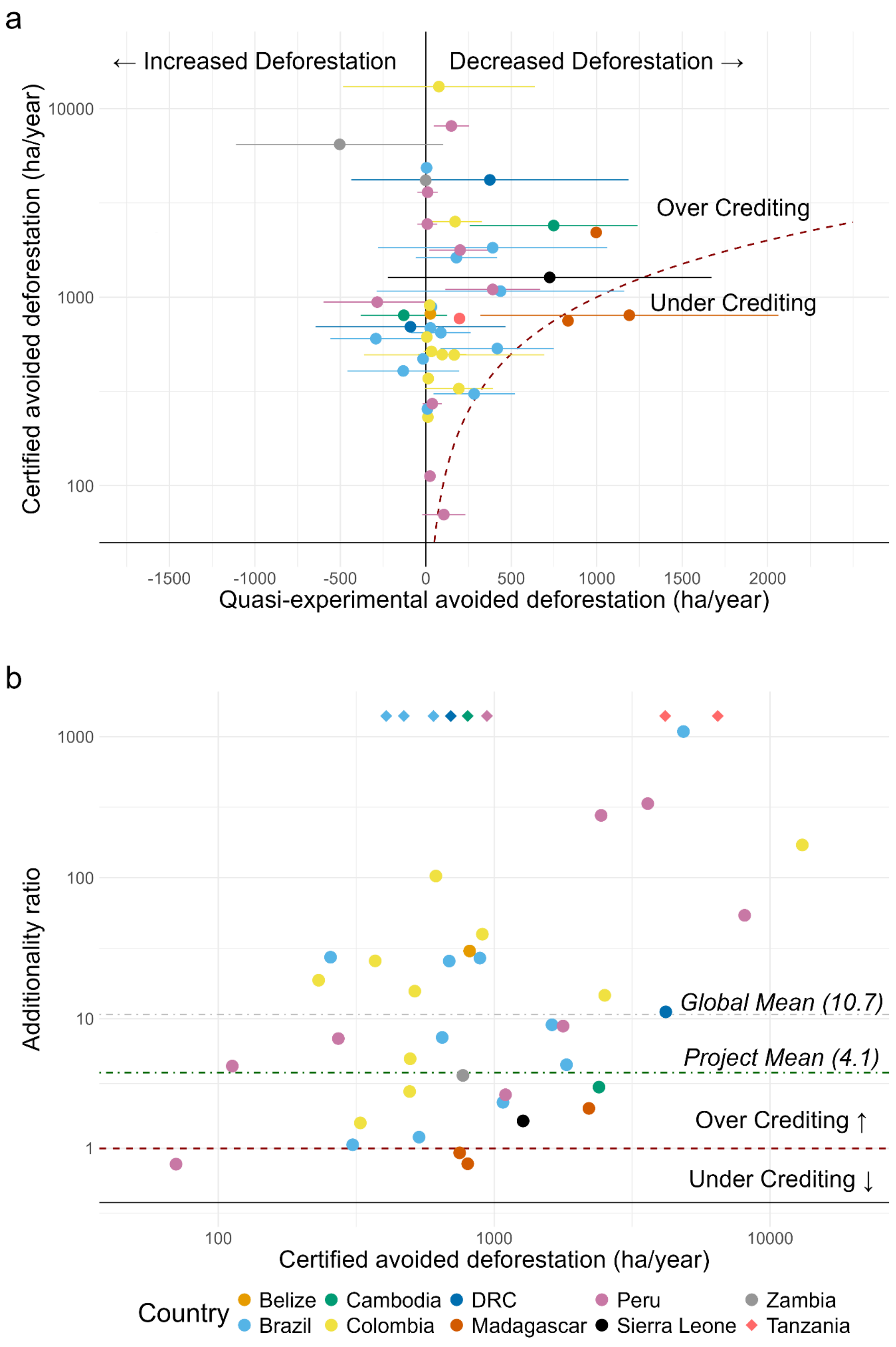

Supplementary figure 2. Comparison of quasi-experimental and certified estimates of project performance coloured by the country where the project was implemented**.** (a) Quasi-experimental evaluations show consistent evidence that REDD+ projects slowed deforestation, but certified estimates were higher, indicating widespread over-crediting. Points represent mean estimates from the six quasi-experimental studies, with error bars showing 95% confidence intervals. (b) The over-crediting ratio (the certified estimate divided by the mean quasi-experimental estimate) plotted against certified avoided deforestation estimates. The dashed grey line indicates the global mean over-crediting ratio and the dashed red lines mark parity between certified and quasi-experimental estimates. Points

above and below the red line represent over- and under-crediting respectively. Colours denote the country where the project was implemented. The over-crediting ratio is undefined for the points that experienced more deforestation than predicted by their quasi-experimental controls (shown in red). Both panels use log-scaled y-axes.

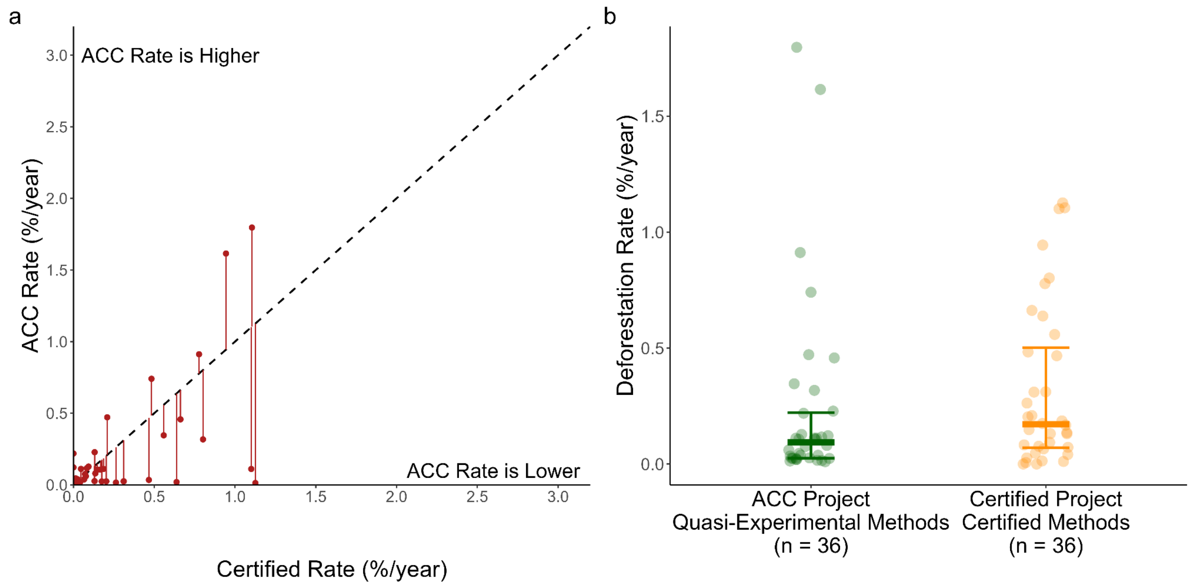

Supplementary figure 3. The remotely sensed deforestation rates in project areas when forest cover is defined as undisturbed or degraded. (a) *Ex post* annual deforestation rates in 36 project areas measured using the European Unions' Annual Change Collection (ACC) versus the bespoke forest cover layers used in certification. The identity line indicates where estimates are equal. (b) Changing the forest cover definition when using the ACC data to include the **undisturbed and degraded** forest classes reduced the *ex post* deforestation rates measured in project areas from 0.26%/year reported in figure 3 of the main text to 0.09%/year. The lower ACC deforestation rates result in there being no difference from the certified project deforestation rates (*Wilcoxon paired signed-ranks,* n = 36, V = 248, p = 0.1866). Error bars show the median and interquartile range. The reason for the reduction in ACC measured deforestation rates is that deforestation is judged to have occurred when disturbances last >2.5 years, even if regrowth subsequently occurs: short duration disturbances (<2.5 years) are not considered.

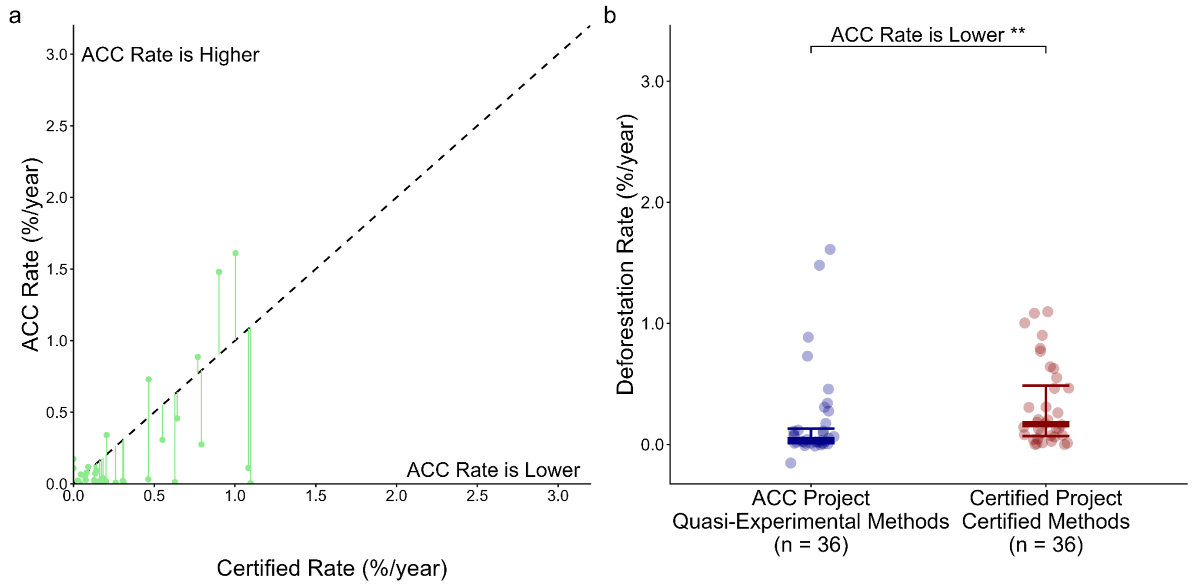

Supplementary figure 4. The remotely sensed deforestation rates in project areas when forest cover is defined as undisturbed, degraded or regrowth. *Ex post* annual deforestation rates in 36 project areas measured using the European Unions' Annual Change Collection (ACC) versus the bespoke forest cover layers used in certification. The identity line indicates where estimates are equal. (b) Changing the forest cover definition when using the ACC data to include the **undisturbed, degraded and regrowth** forest classes reduced the *ex post* deforestation rates measured in project areas from 0.26%/year reported in figure 3 of the main text to 0.04%/year. The ACC deforestation rates measured like this are significantly lower than the certified project deforestation rates (*Wilcoxon paired signed-ranks,* n=36 V = 173, p = 0.01098). Error bars show the median and interquartile range. The reduction in ACC measured deforestation rates are again lower because deforestation is judged to have occurred when disturbances last >2.5 years: short duration disturbances (<2.5 years) are not considered and even longer term disturbances are not considered if regrowth subsequently occurs.

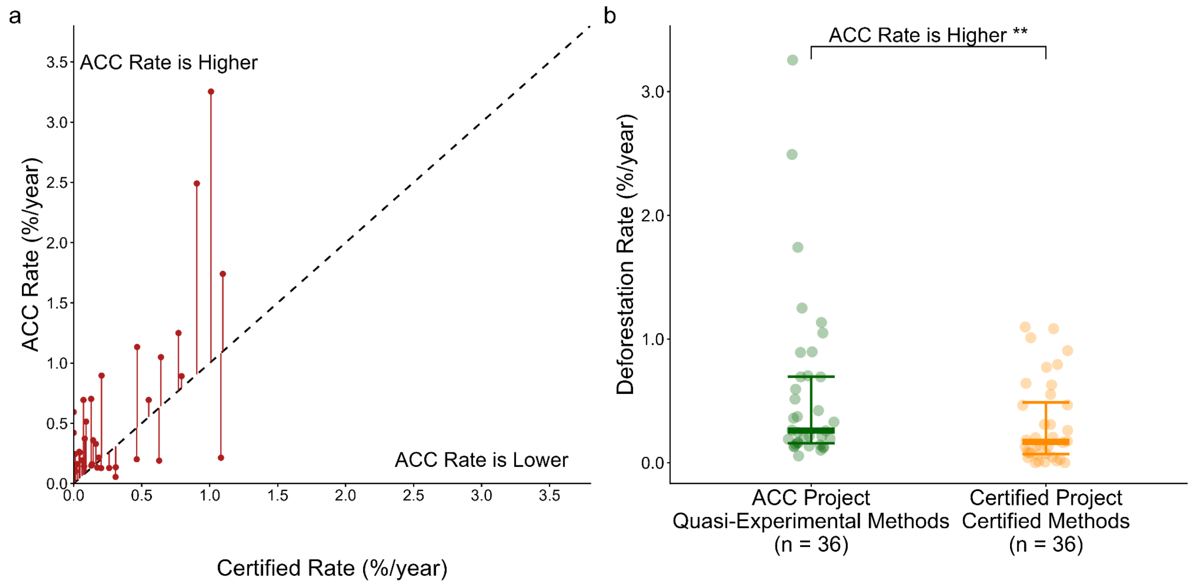

Supplementary figure 5. The remotely sensed deforestation rates in project areas when deforestation is defined as loss of undisturbed or degraded ACC classes. *Ex post* annual deforestation rates in 36 project areas measured using the European Unions' Annual Change Collection (ACC) versus the bespoke forest cover layers used in certification. The identity line indicates where estimates are equal. (b) Measuring deforestation as transitions: (1) from the undisturbed class to any other class, or (2) from the degraded class to the deforested class, did not change the *ex post* deforestation rates measured in project areas when compared just measuring the loss of undisturbed. The median ACC rate remained at 0.26% a year. Though the median project was relatively unaffected, some projects were more affected than others. For example, deforestation rates in project 1650 rose from 2.95% a year to 3.25% a year. Error bars show the median and interquartile range.

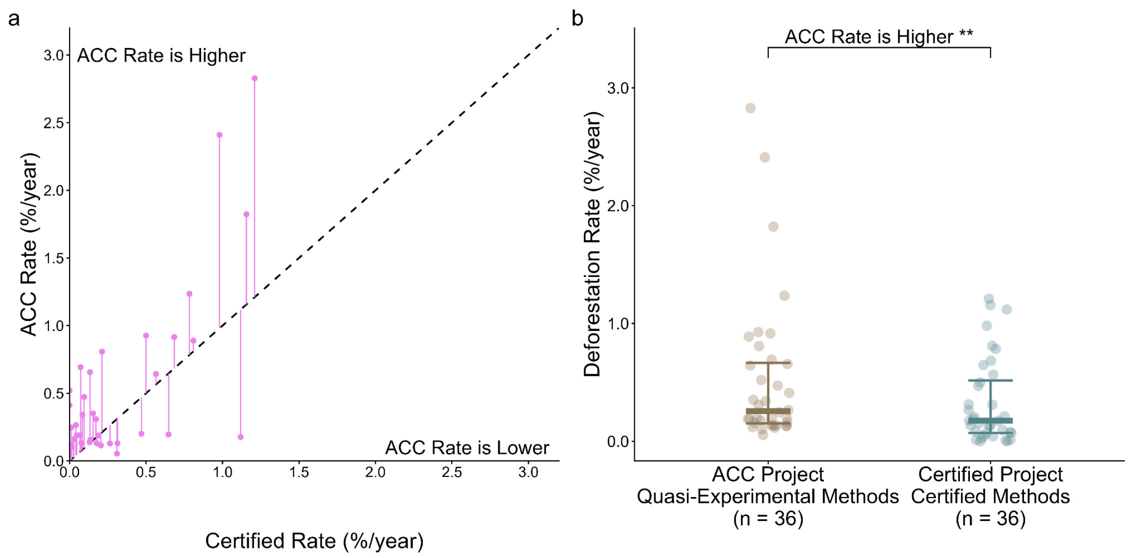

Supplementary figure 6. The remotely sensed deforestation rates in project areas when deforestation is defined as loss of undisturbed or regrowth ACC classes. *Ex post* annual deforestation rates in 36 project areas measured using the European Unions' Annual Change Collection (ACC) versus the bespoke forest cover layers used in certification. The identity line indicates where estimates are equal. (b) Measuring deforestation as transitions: (1) from the undisturbed class to any other class, or (2) from the regrowth class to the deforested class, did not change the median *ex post* deforestation rates measured in project areas when compared with measuring loss of undisturbed forest alone. The median ACC rate remained at 0.26% a year, as presented in figure 3. Error bars show the median and interquartile range.

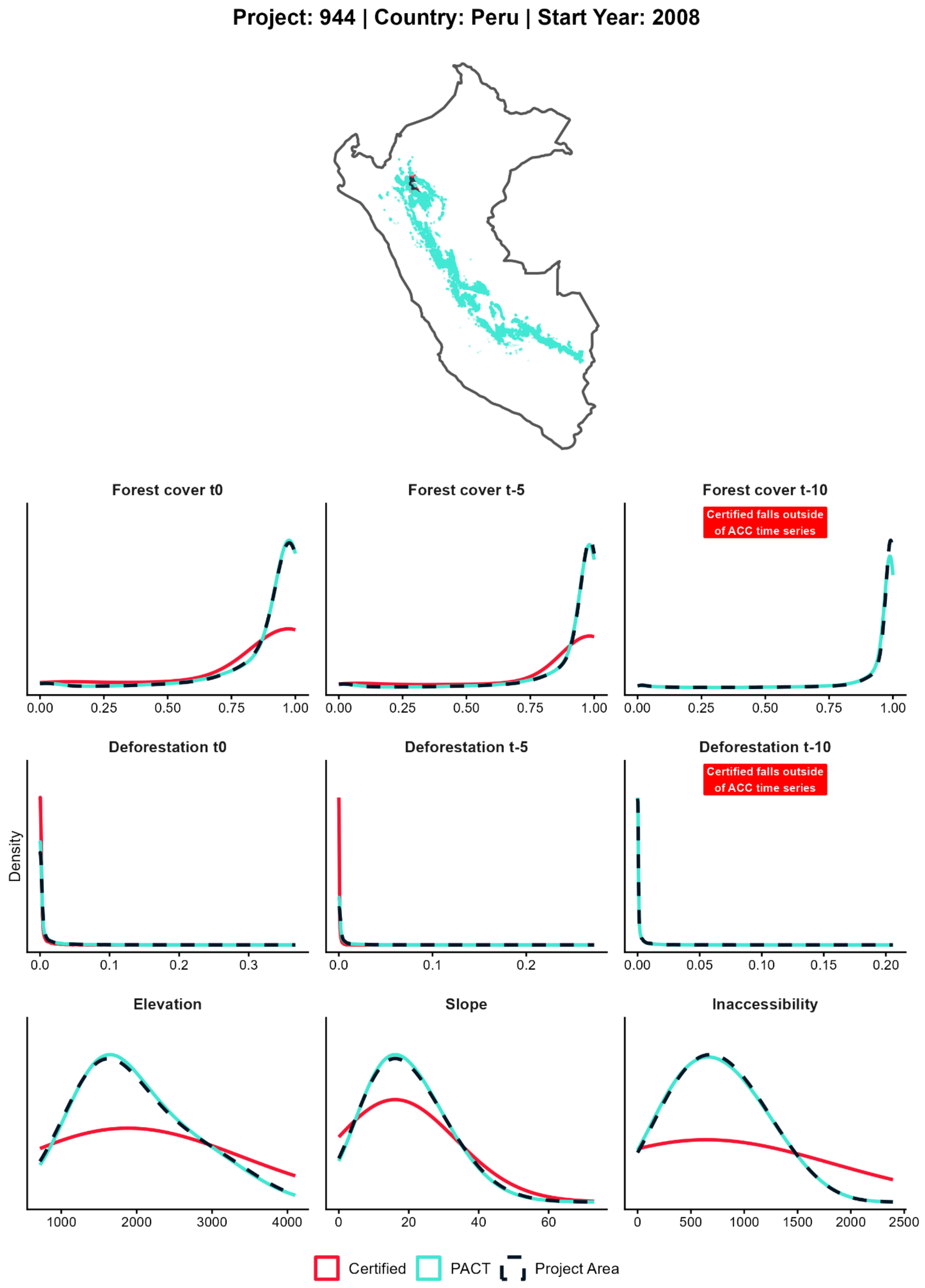

Supplementary figure 7. The location of the project, certified reference and PACT control areas, and differences in exposure to deforestation drivers for project 944**.** Univariate frequency distributions contrast exposure to observable confounders which may influence deforestation. The timing of measurements is expressed relative to the project start year (t_0) for quasi-experimental control areas, or the start of the reference period for reference areas.

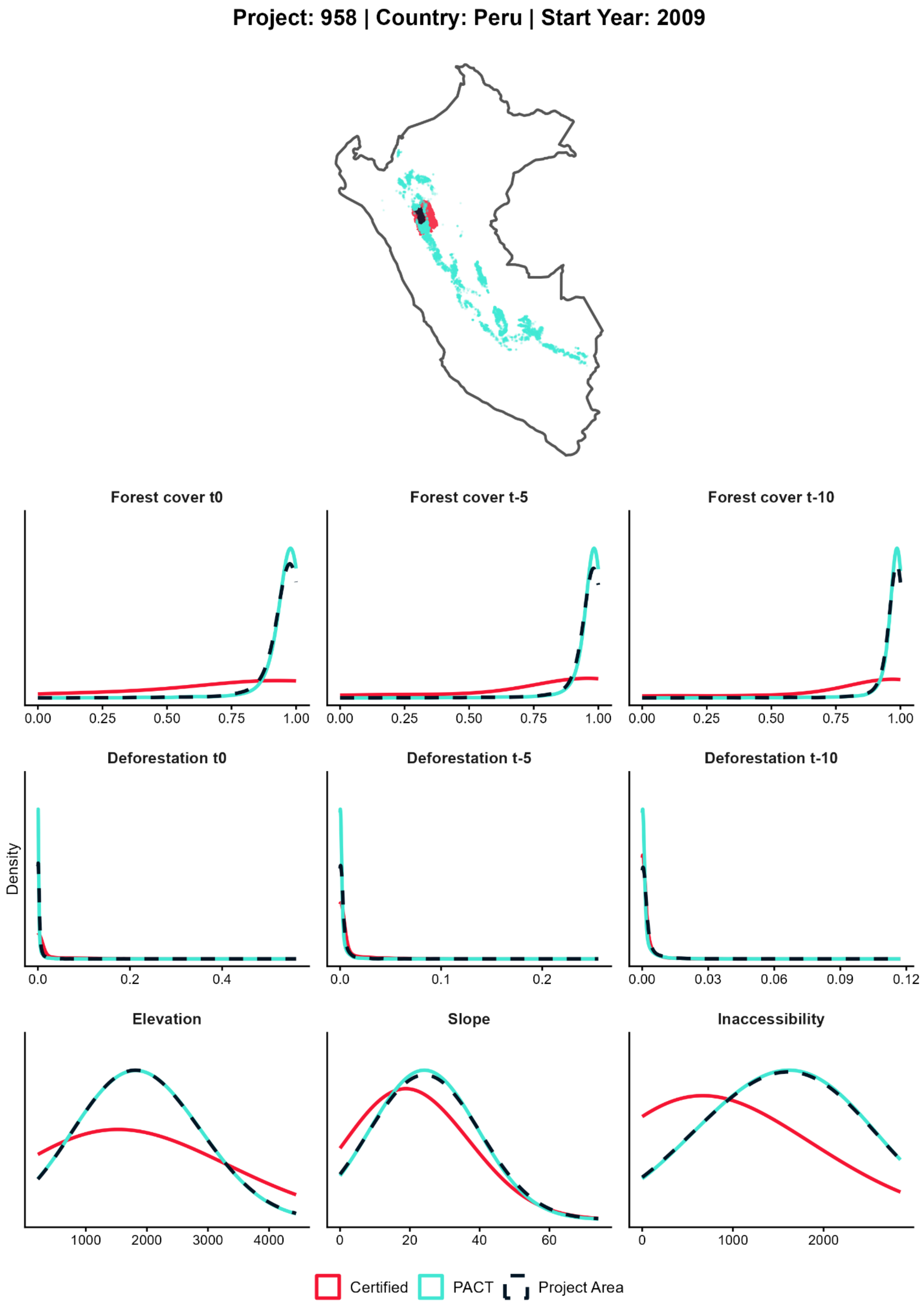

Supplementary figure 8. The location of the project, certified reference and PACT control areas, and differences in exposure to deforestation drivers for project 958**.** Univariate frequency distributions contrast exposure to observable confounders which may influence deforestation. The timing of measurements is expressed relative to the project start year (t_0) for quasi-experimental control areas, or the start of the reference period for reference areas.

Project: 1047 | Country: Madagascar | Start Year: 2007

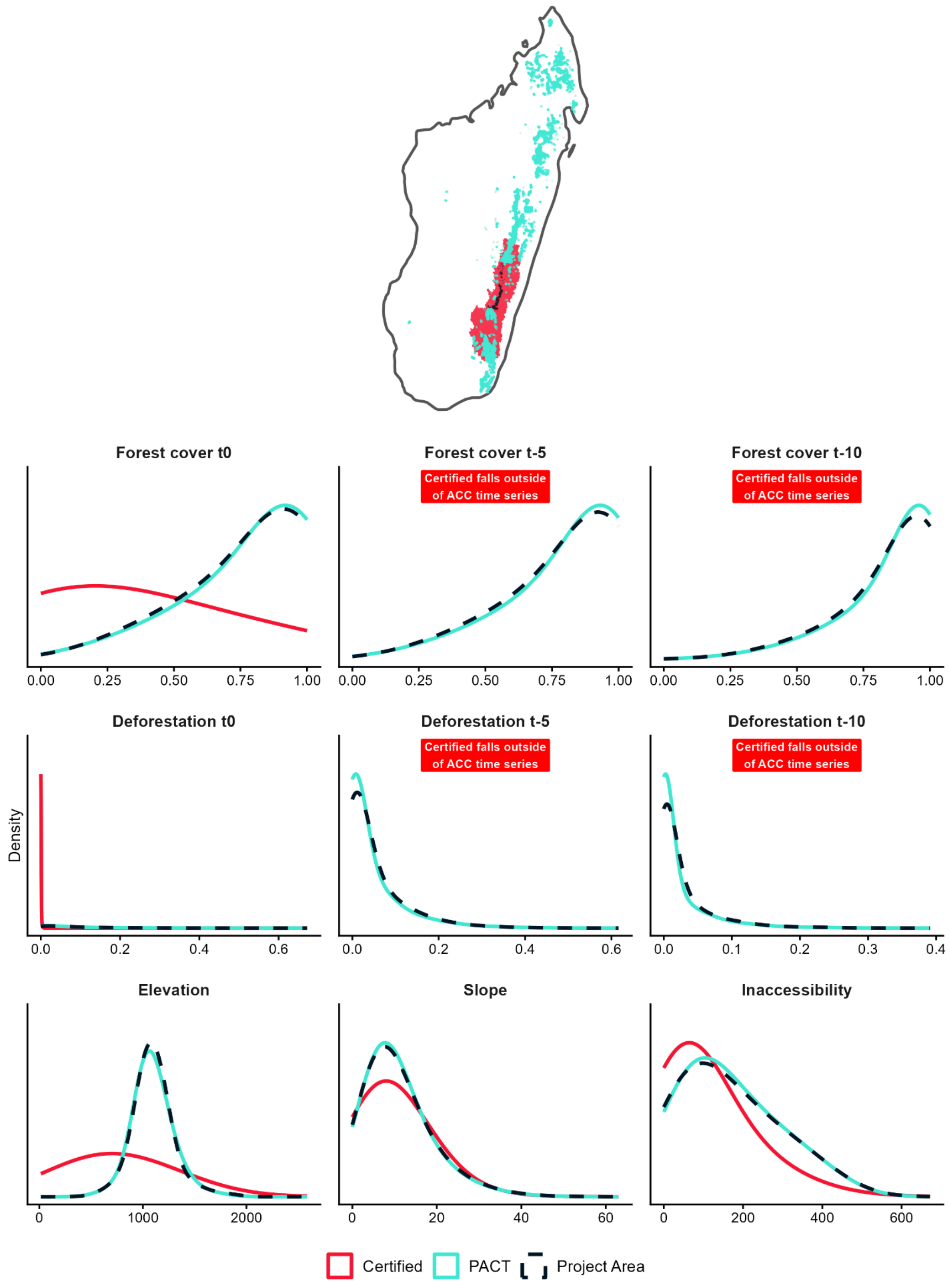

Supplementary figure 9. The location of the project, certified reference and PACT control areas, and differences in exposure to deforestation drivers for project 1047**.** Univariate frequency distributions contrast exposure to observable confounders which may influence deforestation. The timing of measurements is expressed relative to the project start year (t_0) for quasi-experimental control areas, or the start of the reference period for reference areas.

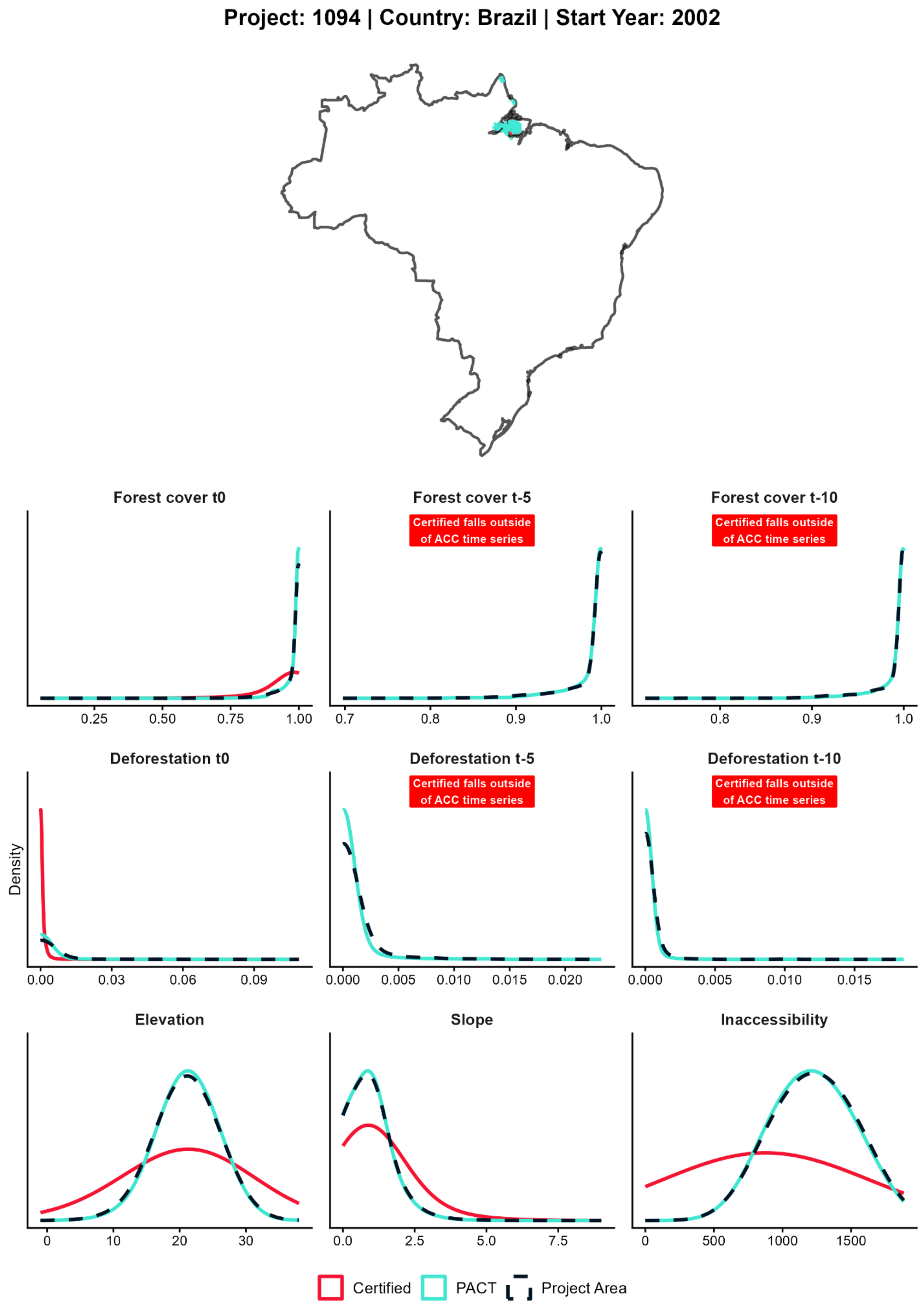

Supplementary figure 10. The location of the project, certified reference and PACT control areas, and differences in exposure to deforestation drivers for project 1094**.** Univariate frequency distributions contrast exposure to observable confounders which may influence deforestation. The timing of measurements is expressed relative to the project start year (t_0) for quasi-experimental control areas, or the start of the reference period for reference areas.

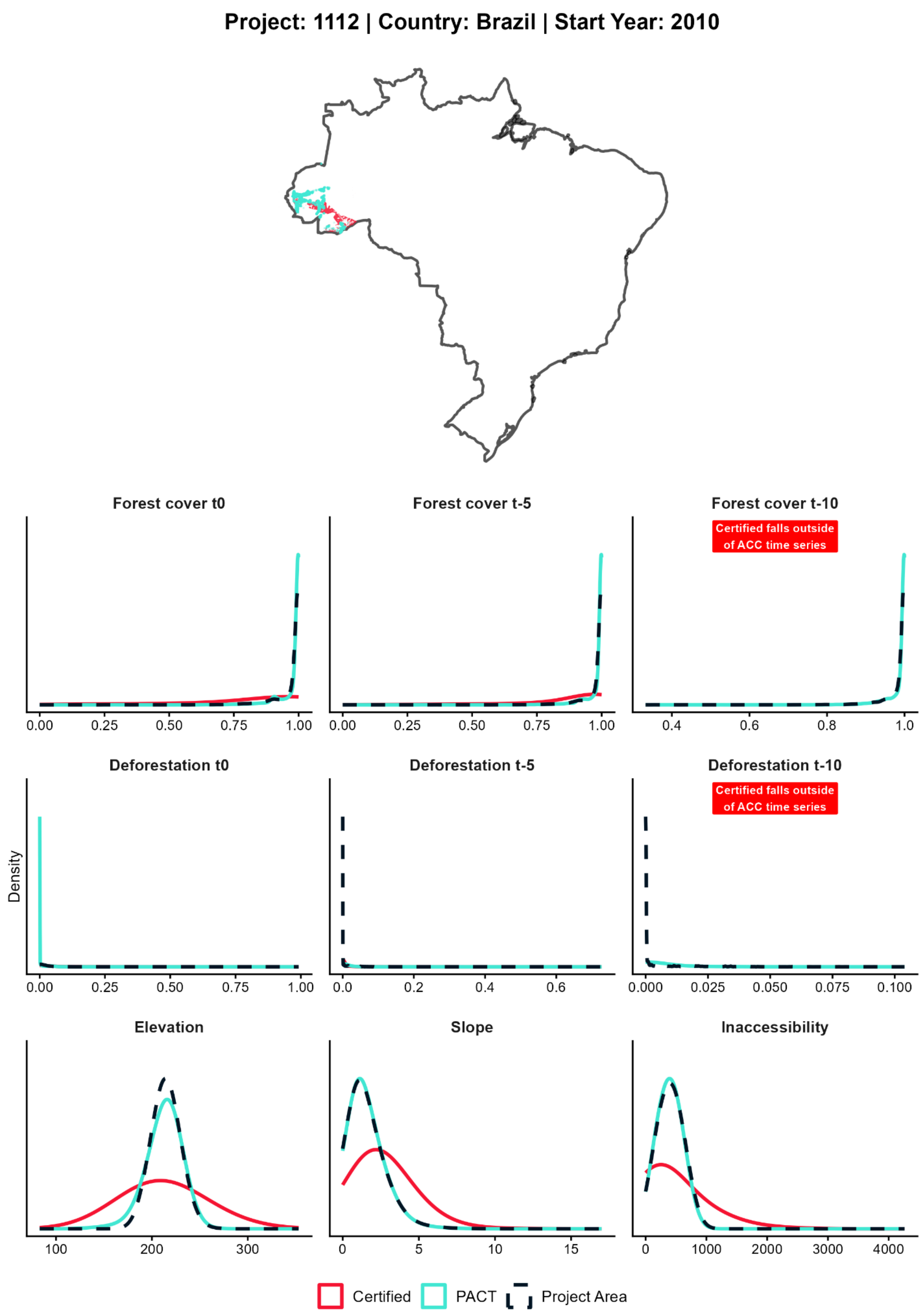

Supplementary figure 11. The location of the project, certified reference and PACT control areas, and differences in exposure to deforestation drivers for project 1112**.** Univariate frequency distributions contrast exposure to observable confounders which may influence deforestation. The timing of measurements is expressed relative to the project start year (t_0) for quasi-experimental control areas, or the start of the reference period for reference areas.

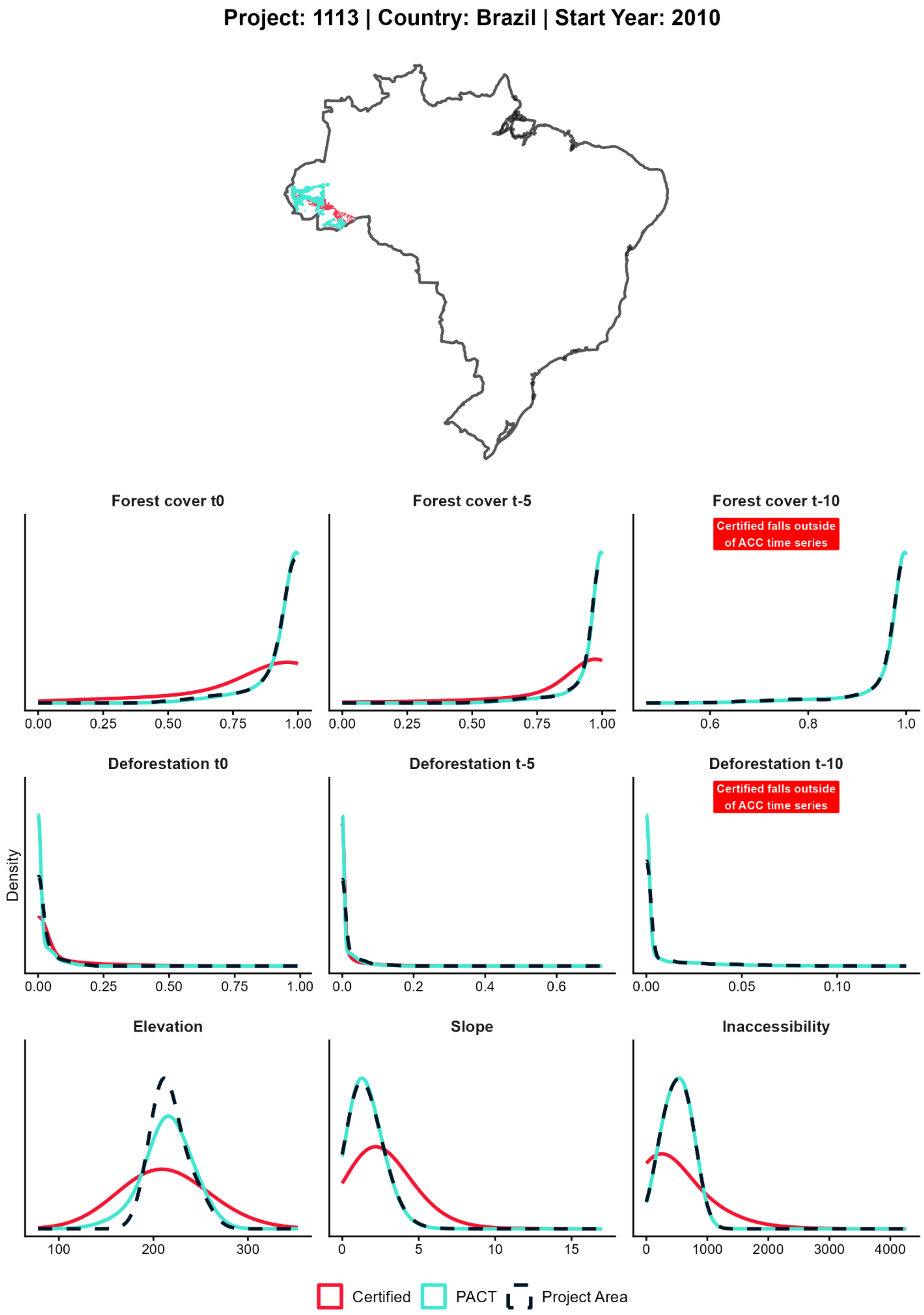

Supplementary figure 12. The location of the project, certified reference and PACT control areas, and differences in exposure to deforestation drivers for project 1113**.** Univariate frequency distributions contrast exposure to observable confounders which may influence deforestation. The timing of measurements is expressed relative to the project start year (t_0) for quasi-experimental control areas, or the start of the reference period for reference areas.

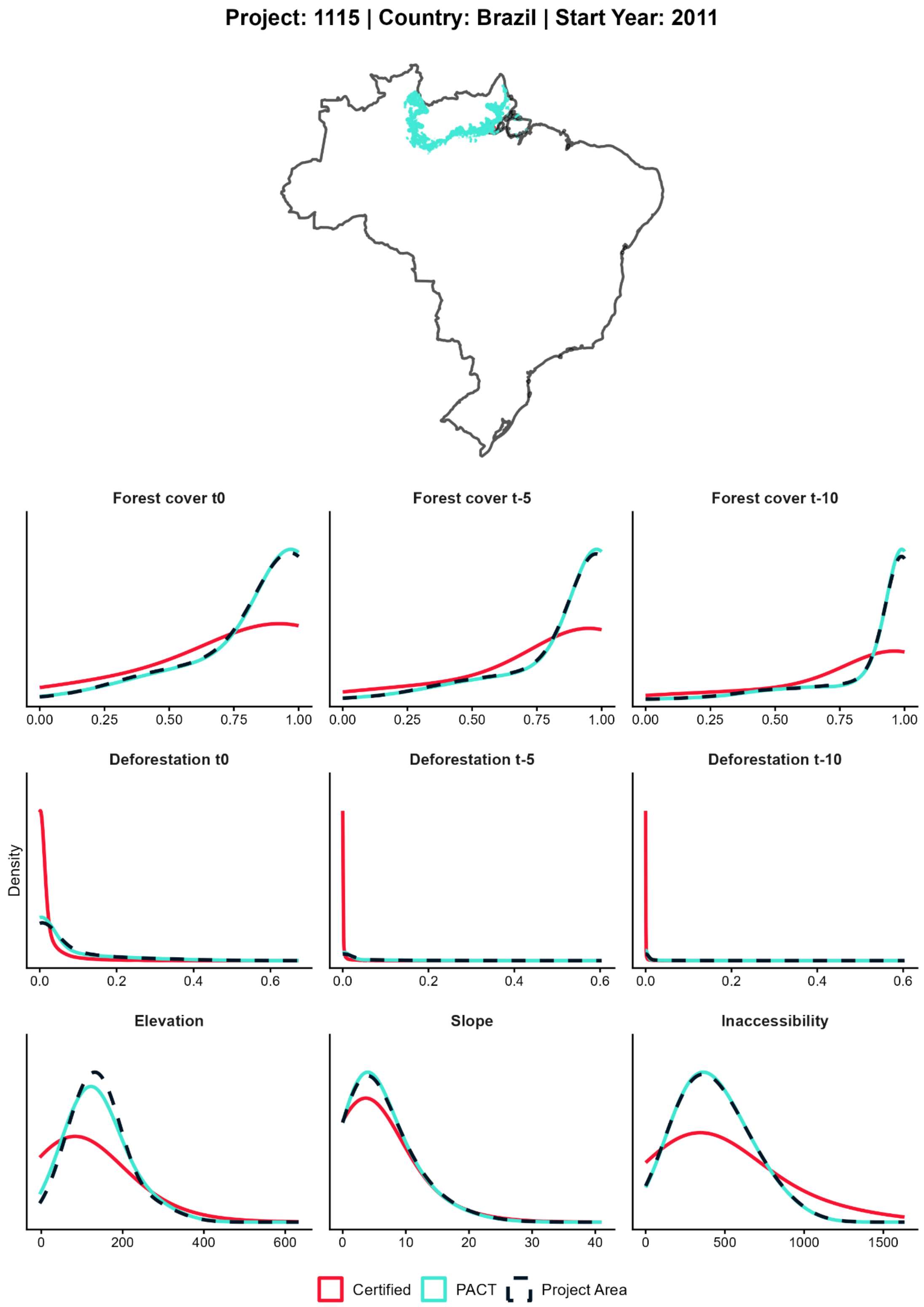

Supplementary figure 13. The location of the project, certified reference and PACT control areas, and differences in exposure to deforestation drivers for project 1115**.** Univariate frequency distributions contrast exposure to observable confounders which may influence deforestation. The timing of measurements is expressed relative to the project start year (t_0) for quasi-experimental control areas, or the start of the reference period for reference areas.

Project: 1201 | Country: Sierra Leone | Start Year: 2012

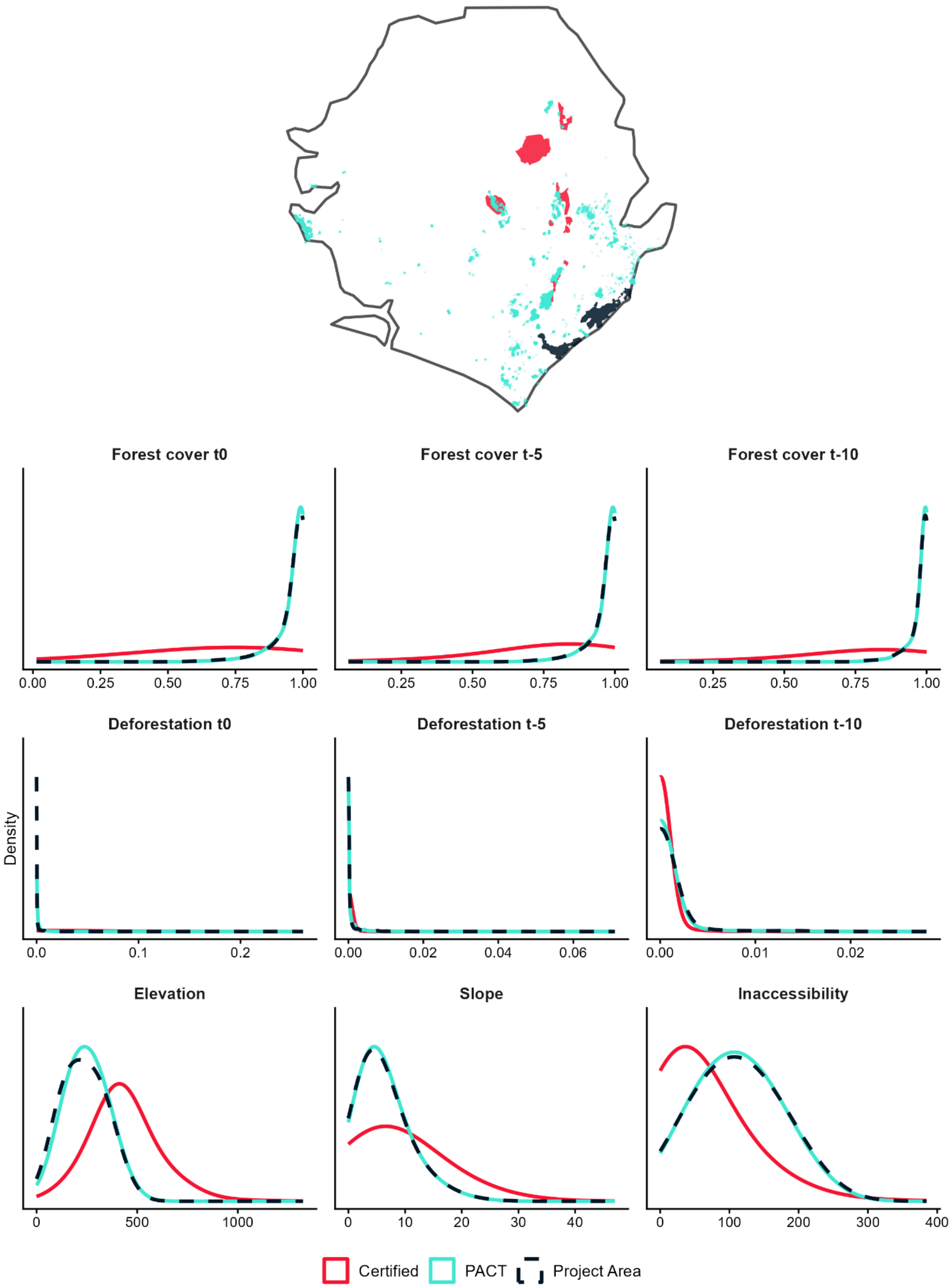

Supplementary figure 14. The location of the project, certified reference and PACT control areas, and differences in exposure to deforestation drivers for project 1201**.** Univariate frequency distributions contrast exposure to observable confounders which may influence deforestation. The timing of measurements is expressed relative to the project start year (t_0) for quasi-experimental control areas, or the start of the reference period for reference areas.

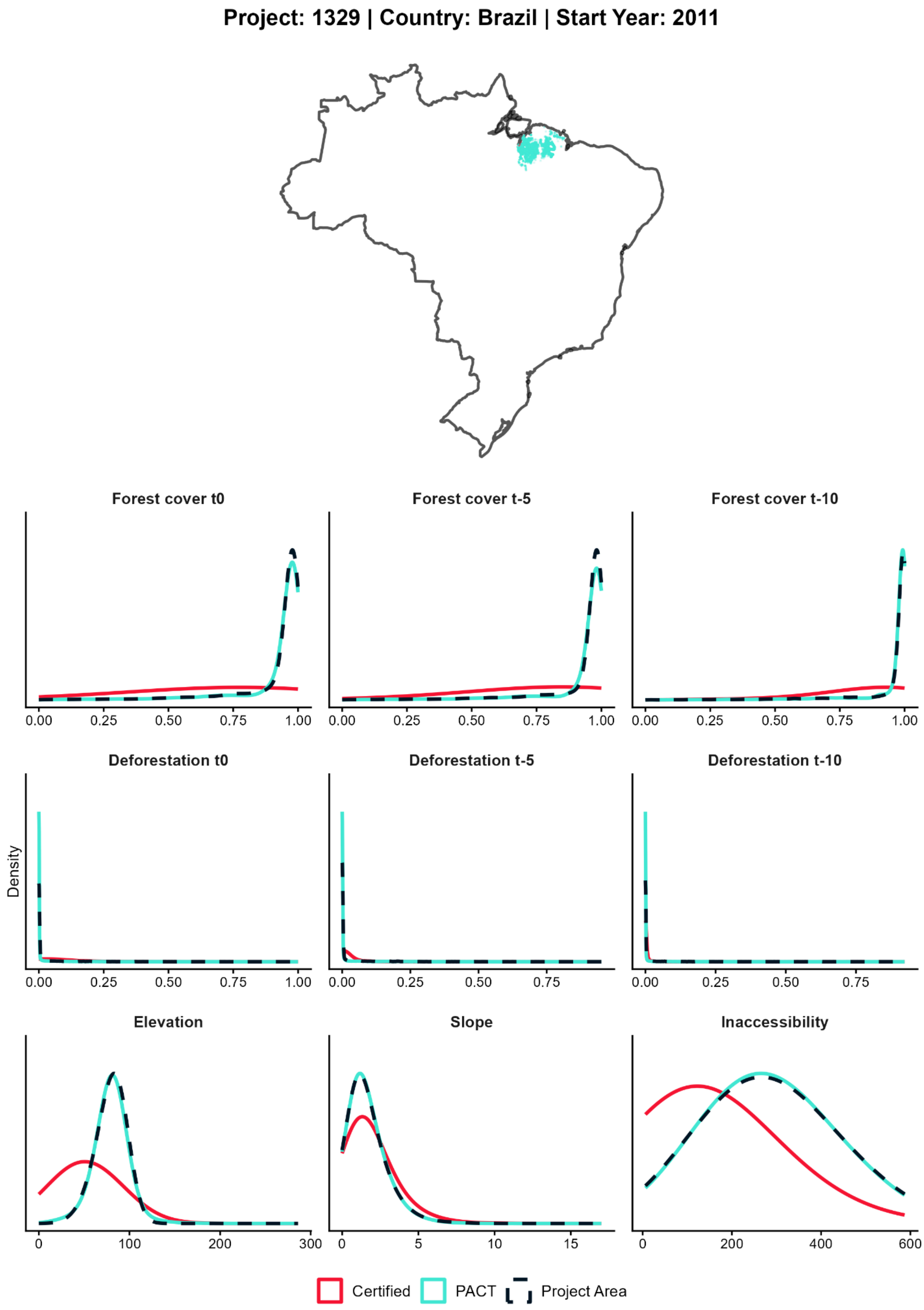

Supplementary figure 15. The location of the project, certified reference and PACT control areas, and differences in exposure to deforestation drivers for project 1329**.** Univariate frequency distributions contrast exposure to observable confounders which may influence deforestation. The timing of measurements is expressed relative to the project start year (t_0) for quasi-experimental control areas, or the start of the reference period for reference areas.

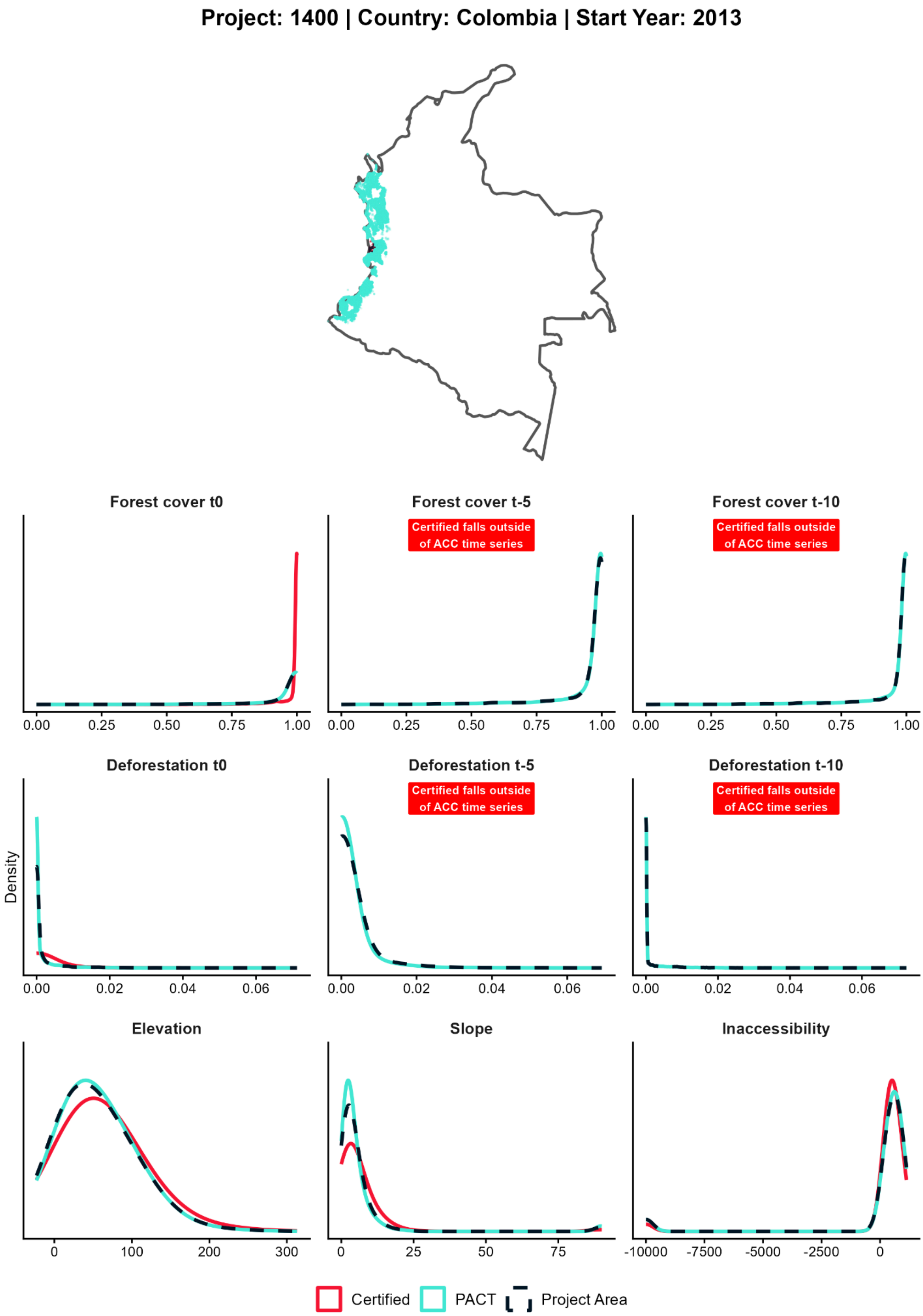

Supplementary figure 16. The location of the project, certified reference and PACT control areas, and differences in exposure to deforestation drivers for project 1400**.** Univariate frequency distributions contrast exposure to observable confounders which may influence deforestation. The timing of measurements is expressed relative to the project start year (t_0) for quasi-experimental control areas, or the start of the reference period for reference areas.

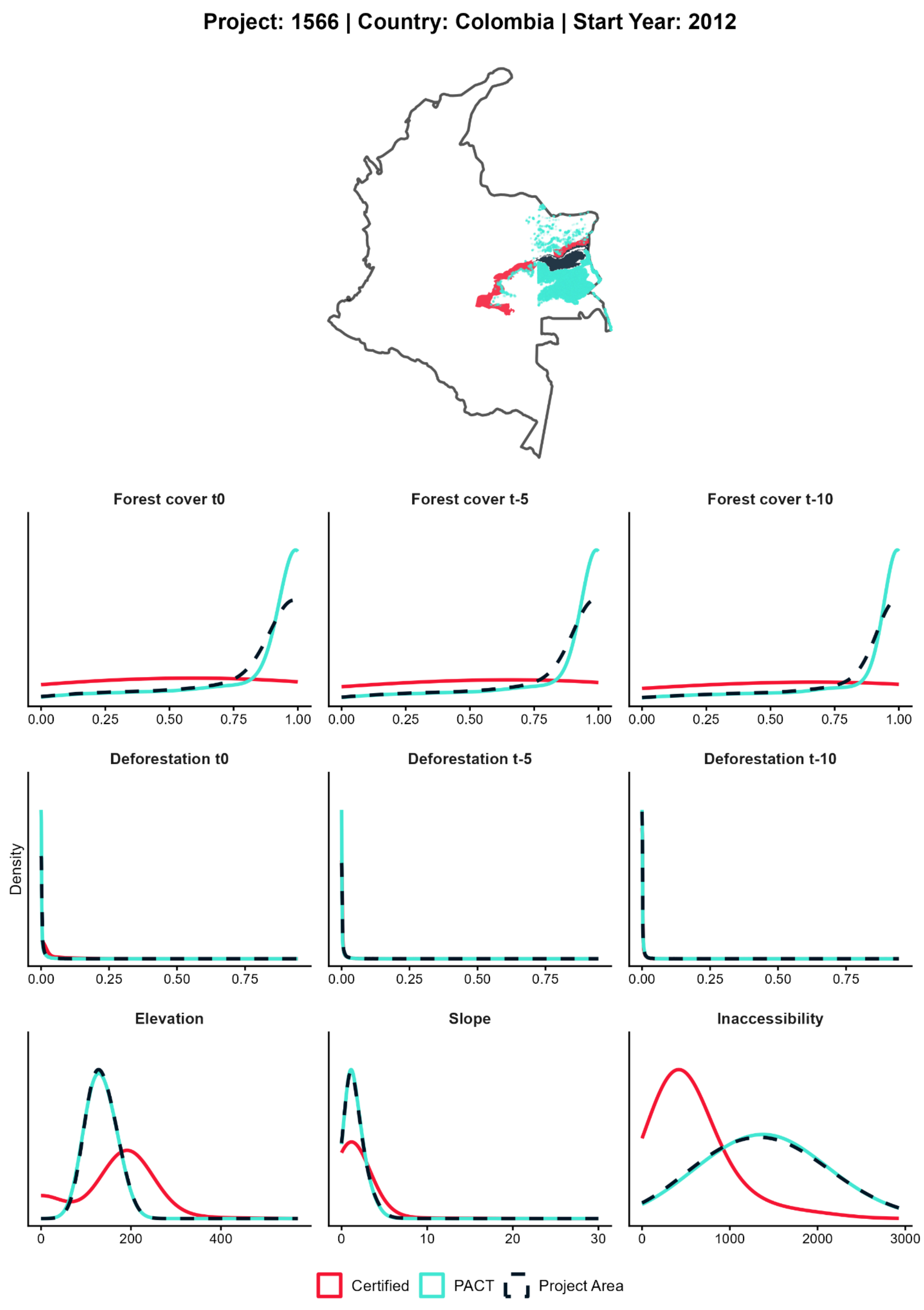

Supplementary figure 17. The location of the project, certified reference and PACT control areas, and differences in exposure to deforestation drivers for project 1566**.** Univariate frequency distributions contrast exposure to observable confounders which may influence deforestation. The timing of measurements is expressed relative to the project start year (t_0) for quasi-experimental control areas, or the start of the reference period for reference areas.

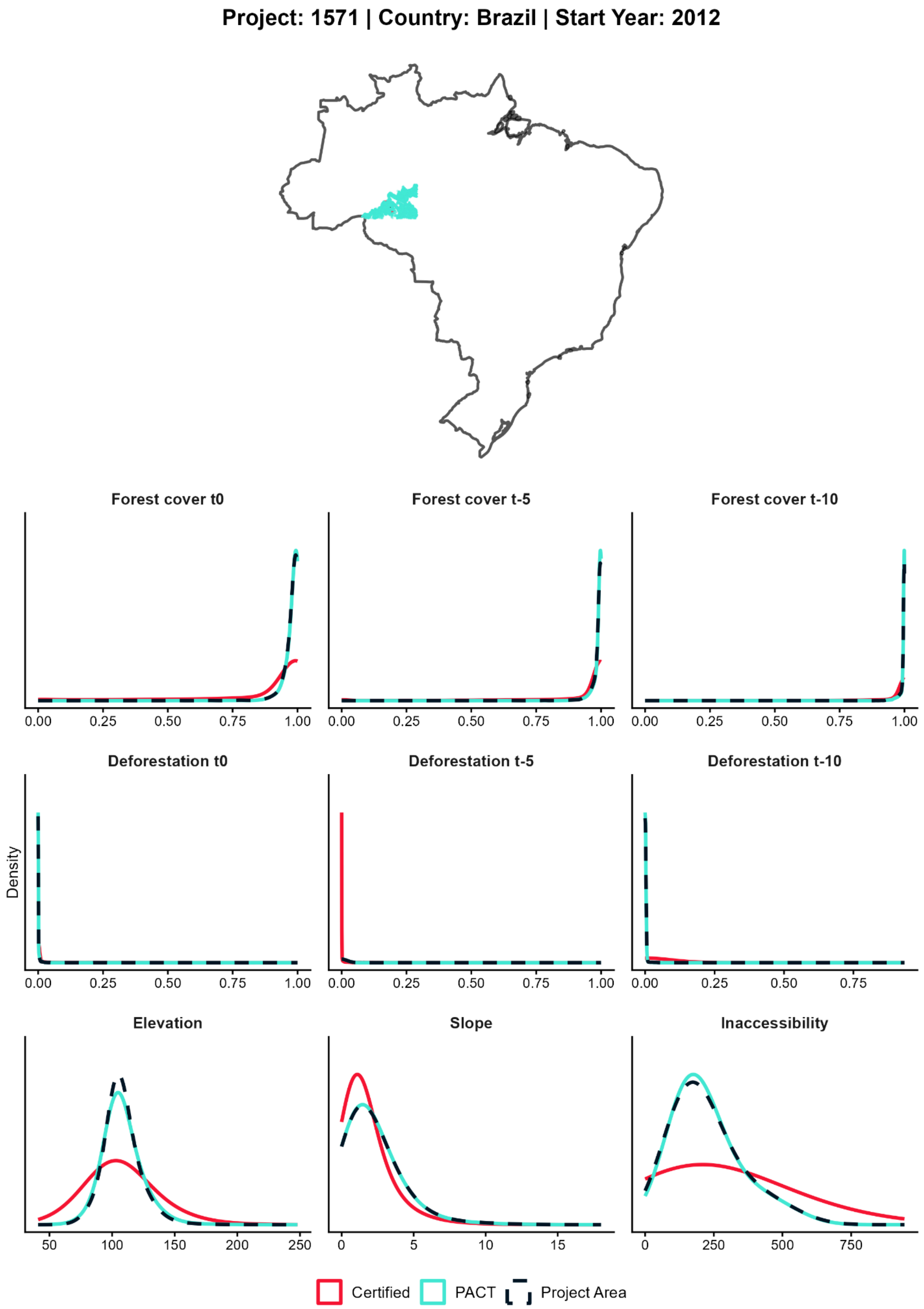

Supplementary figure 18. The location of the project, certified reference and PACT control areas, and differences in exposure to deforestation drivers for project 1571**.** Univariate frequency distributions contrast exposure to observable confounders which may influence deforestation. The timing of measurements is expressed relative to the project start year (t_0) for quasi-experimental control areas, or the start of the reference period for reference areas.

Project: 1650 | Country: Cambodia | Start Year: 2009

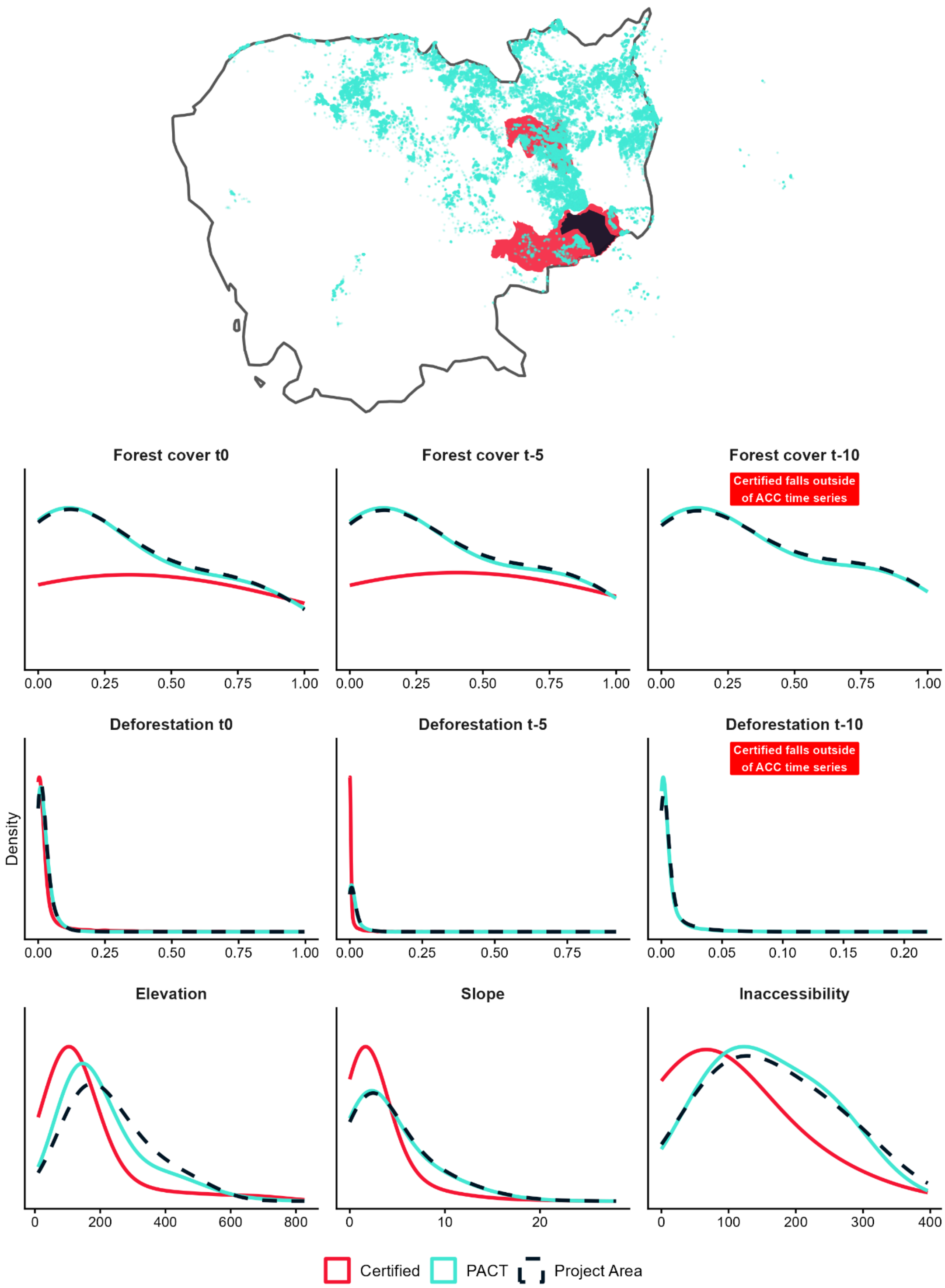

Supplementary figure 19. The location of the project, certified reference and PACT control areas, and differences in exposure to deforestation drivers for project 1650**.** Univariate frequency distributions contrast exposure to observable confounders which may influence deforestation. The timing of measurements is expressed relative to the project start year (t_0) for quasi-experimental control areas, or the start of the reference period for reference areas.

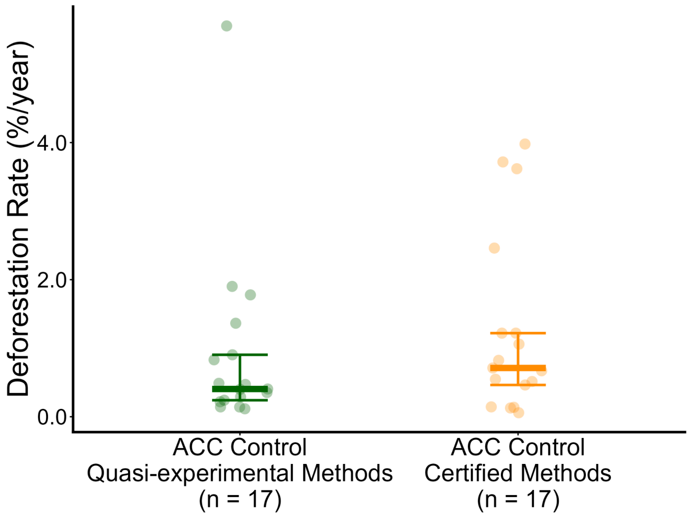

Supplementary figure 20. The remotely sensed deforestation rates in control areas when deforestation is defined as loss of undisturbed or degraded. Measuring deforestation as transitions: (1) from the undisturbed class to any other class, or (2) from the degraded class to the deforested class, had no effect on the median deforestation rate measured in quasi-experimental control areas, which remained at 0.41%/year, as presented in figure 4. In certified control areas deforestation rates increased from 0.69%/year in figure 4 to 0.71%/year. Error bars show the median and interquartile range.

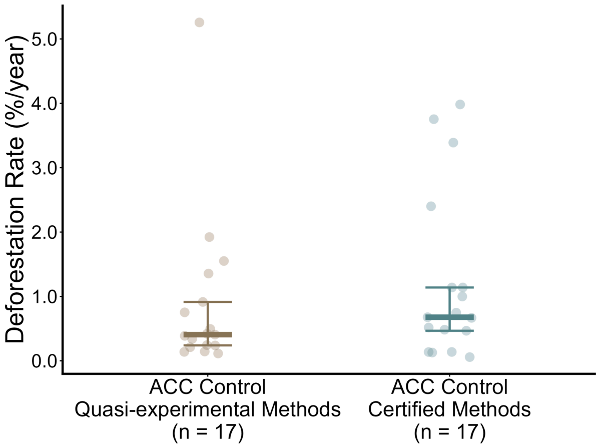

Supplementary figure 21. The remotely sensed deforestation rates in control areas when deforestation is defined as loss of undisturbed or regrowth. Measuring deforestation as transitions: (1) from undisturbed forest to any other class, or (2) from the regrowth class to the deforested class, had no effect on the median deforestation rate measured in quasi-experimental control areas, which remained at 0.41%/year, as presented in figure 4. In certified control areas deforestation rates decreased from 0.69%/year in figure 4 to 0.68%/year. Error bars show the median and interquartile range.

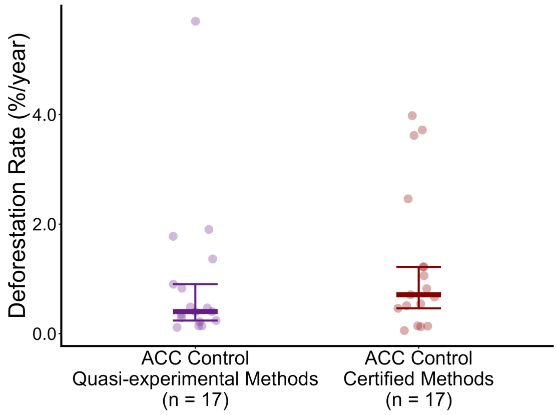

Supplementary figure 22. The remotely sensed deforestation rates in control areas when deforestation is defined as loss of undisturbed, degraded or regrowth. Measuring deforestation as transitions: (1) from the undisturbed class to any other class, (2) from the degraded class to the deforested class, or (3) from the regrowth class to the deforested class, had no effect on the median deforestation rate measured in quasi-experimental control areas, which remained at 0.41%/year, as presented in figure 4. In certified control areas deforestation rates increased from 0.69%/year in figure 4 to 0.71%/year. Error bars show the median and interquartile range.

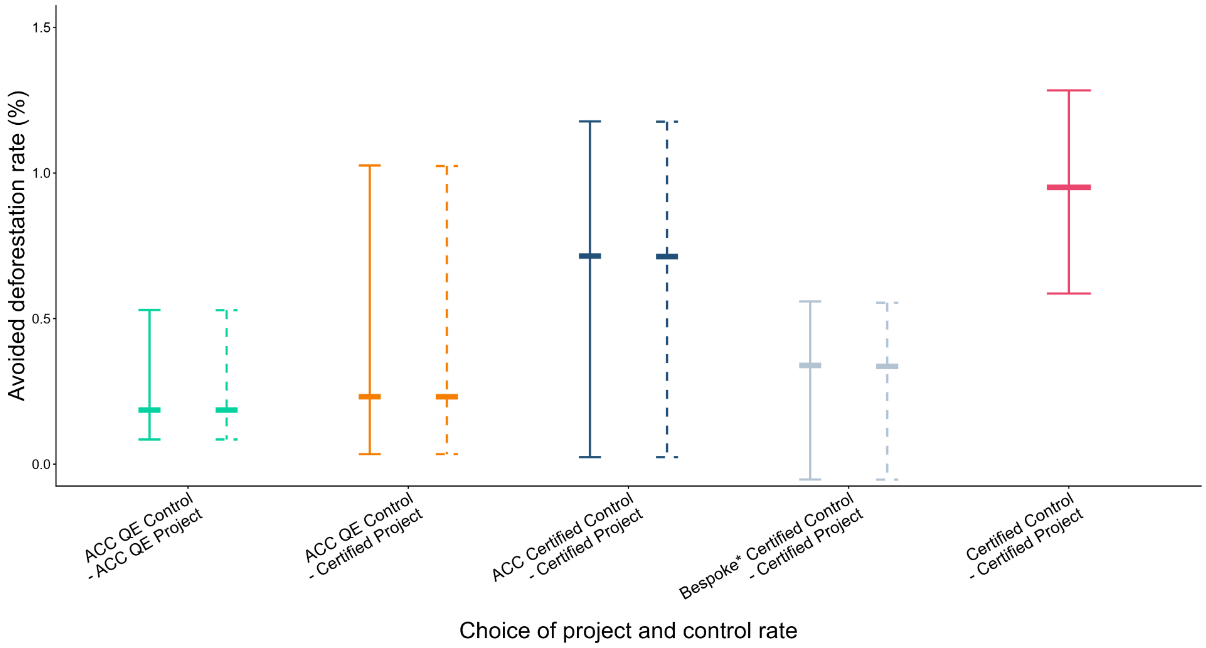

Supplementary figure 23. The avoided deforestation rates for the 17 projects in the main analysis (solid lines in Fig. 5) are compared with avoided deforestation rates that also include transitions from the regrowth class to the deforested class (dashed lines). Almost no differences were observed due to the very low proportion of regrowth pixels within projects. There is no bar for deforestation rates including regrowth for certified control - certified project, as this does not make use of the ACC layers. Error bars represent medians and interquartile ranges.

Supplementary note 2. Differences in avoided deforestation estimates produced by alternative certified modelling approaches.

For the first generation of REDD+ projects, there were several different certification methods available for estimating counterfactual deforestation. West, Bomfim, and Haya [2] applied the four most common Verra methods (Table 1 in the main text) to four projects to assess the sensitivity of avoided deforestation estimates in control areas to the choice of the four most common certification methods. Within these 4 methods, the impact of different modelling configurations was also explored: for VM0006 the forest scarcity factor was applied at two separate levels, and for VM0007 and VM0015 two different algorithms were used to develop spatial deforestation risk maps. This resulted in two estimates for each of these three methods. Thus, there were a total of eight separate estimates across the four methods, including the projects' certified estimate. Taking West, Bomfim, and Haya's estimates, we contrasted the outcomes of various certification methods against the distribution of different quasi-experimental results using t-tests.

$$p_{\mathrm{m}} = 1 - F_t\left(|t_{\mathrm{m}}|, df_{\mathrm{q}}\right) \quad \text{Eq. 1}$$

$$t_{\mathrm{m}} = \frac{x_{\mathrm{m}} - \mu_{\mathrm{q}}}{\mathrm{SE}_{\mathrm{q}}} \quad \text{Eq. 2}$$

We extracted the mean (μ_{q}) and standard error (SE_{q}) of quasi-experimental estimates of the avoided deforestation achieved by each of the four projects. Using these we calculated the one tailed t-scores (t_{m}) of each certification method estimate (x_{m}) against the quasi-experimental distribution. With these t-scores and the degrees of freedom allotted by the number of quasi-experimental findings for each project (df_{q}) we calculated p-values for the estimates produced by each certified method (p_{m}) across the respective cumulative probability function of the t-distribution (F_t). These p-scores represent the probability that an estimate produced by a certification method was significantly higher than the distribution of quasi-experimental findings (in other words a result that could be considered an example of over-crediting).

Modelling approaches produced a range of avoided deforestation estimates. Comparing these with the quasi-experimental estimates revealed that projects each could have produced a deforestation estimate within, or more conservative than, the distribution of quasi-experimental estimates. However, for 3 of 4 projects, modelling approaches generated estimates significantly higher than these distributions. For these projects, the method used for certification produced one of these higher estimates. As an example, 5 of the 8 possible estimates for Project 944 were similar to the distribution of quasi-experimental results (Table 2). Yet, the modelling approach chosen produced a significantly higher-than-probable (t = 18.25, d.f. = 3, p = 0.0002) estimate of 1777.7 ha/year of avoided deforestations (against the quasi-experimental mean of 139.1 ha/year). In the case of Project 1396, the only example

where the certified estimate was not significantly different from quasi-experimental results, all possible alternatives fell within the quasi-experimental distribution.

Supplementary table 2. Modelling approaches, available under different certification methods, typically produced higher estimates of avoided deforestation than quasi-experimental methods, and projects used methods which produced improbably high estimates. Estimates of avoided deforestation (ha/year) for four projects, based on four quasi-experimental methods (QEM) applied by independent researchers, against the certified estimates used to produce credits, and alternative estimates from different possible certification methods by West, Bomfim and Haya[10]. The methods used are summarised in table 1. Significant differences between certified estimates and the mean and distribution of quasi-experimental results were assessed using t-tests, with *t*-scores reported. Estimates above the 95% confidence intervals for quasi-experimental method (QEM) results are shaded in grey, with significance indicated by * p<0.05, ** p<0.01, and *** p<0.001. No adjustment was made for multiple testing.

**Method**	***Project 934***	***Project 944***	***Project 1112***	***Project 1396***
QEM n	3	4	4	4
QEM Mean	180.8	139.1	78.4	378.6
QEM 95% CI	-853.0 to 1214.6	-13.3 to 291.5	-13.1 to 170.0	1.1 to 756.1
Certified Estimate	4815.8 ** (T = 7.20, p = 0.0094)	1750.4 *** (T = 17.68, p < 0.001)	674.8 *** (T = 11.18, p < 0.001)	1210.2 * (T = 3.74, p = 0.0167)
VM0006-FSF-1	-1589.2 (T = -3.06, p = 0.9539)	-48.1 (T = -2.11, p = 0.9370)	2.1 (T = -1.53, p = 0.8882)	54.6 (T = -1.46, p = 0.8794)
VM0006-FSF-2	-1691.6 (T = -3.23, p = 0.9579)	-107.9 (T = -2.76, p = 0.9650)	-6.5 (T = -1.69, p = 0.9054)	39.6 (T = -1.52, p = 0.8876)
VM0007-MLP	3185.3 * (T = 4.59, p = 0.0222)	-262.5 (T = -4.46, p = 0.9895)	5415.8 *** (T = 100.74, p < 0.001)	404.0 (T = 0.11, p = 0.4582)
VM0007-SW	4123.2 * (T = 6.09, p = 0.0129)	3062.2 *** (T = 32.10, p < 0.001)	24.9 (T = -1.10, p = 0.8237)	190.8 (T = -0.84, p = 0.7698)
VM0009	519.3 (T = 0.32, p = 0.3903)	7530.3 *** (T = 81.25, p < 0.001)	77.1 (T = -0.11, p = 0.5414)	179.7 (T = -0.89, p = 0.7815)
VM0015-MLP	3140.8 * (T = 4.52, p = 0.0228)	-232.8 (T = -4.14, p = 0.9872)	5153.6 *** (T = 95.78, p < 0.001)	402.4 (T = 0.11, p = 0.4609)
VM0015-SW	4077.8 * (T = 6.02, p = 0.0132)	2779.3 *** (T = 28.99, p < 0.001)	22.3 (T = -1.15, p = 0.8328)	189.9 (T = -0.85, p = 0.7708)

Supplementary table 3. Details of the calculation of avoided deforestation using the numbers extracted from the most recent VERRA monitoring reports for each project included in our assessment. Certified numbers were taken from the most recent monitoring reports or project design documents which are publicly available on the VCS search registry. Mean annual deforestation was calculated by summing the total deforestation across the years considered and dividing by the length of the monitoring period, calculated as the difference between the start and end year.

Project ID	Start year	End year	Total certified counterfactual deforestation (ha)	Total certified project deforestation (ha)	Total certified avoided deforestation (ha)	Mean Annual Avoided Deforestation (ha/ yr)
844	2009	2018	21,982.83	0.00	21982.83	2442.536
856	2010	2017	1,618	0.00	1618	231.1429
875	2009	2019	18391.23	2185.6	16205.63	1620.563
904	2008	2011	5,444.00	3,039.00	2405	801.6667
934	2011	2021	NA	NA	41889.19	4188.919
944	2009	2020	22,358.00	3,268.00	19555*	1777.727
958	2010	2020	1,525.00	822.00	703	70.3
963	2011	2020	6,037.00	1,801.50	4235.5	470.6111
977	2009	2011	3,698.40	39.70	3658.7	1829.35
981	2009	2017	9681	1069	8612	1076.5
985	2009	2021	98134.45	1040.22	97094.23	8091.186
1047	2007	2012	7,090.00	3,080.00	4010	802
1067	2010	2020	11893	955.15	10993.02**	1099.302
1094	2003	2017	5,469.02	1,895.02	3574	255.2857
1112	2012	2017	3,654.00	217.90	3436.1	687.22
1113	2012	2017	4788.6	349.6	4439	887.8
1115	2011	2019	5957	771	5186	648.25
1122	2009	2012	NA	NA	1908.542	636.1808
1201	2012	2019	9,105.00	194.00	8911	1273
1202	2009	2021	9327.44	80.60	9246.84	770.57
1215	2005	2013	10641	4643	5998	749.75
1218	2011	2020	2,658.68	207.30	2451.38	272.3756

1311	2008	2011	16,299.00	9,684.00	6615	2205
1326	2011	2020	8240	904.9258	7335.074	815.0082
1329	2012	2019	2175	25.25	2149.75	307.1071
1359	2009	2013	NA	NA	2786	696.5
1360	2010	2019	18260.6	9786.4	8474.2	941.5778
1382	2013	2021	NA	NA	38836.2	4854.525
1389	2013	2018	5354	2874	2480	496
1390	2014	2017	1954	472	1482	494
1391	2013	2017	3579	1120	2459	614.75
1392	2013	2017	4514.8917	890	3624.892	906.2229
1395	2013	2017	11,016.16	951.00	10065.16	2516.291
1396	2014	2017	2600	1618	982	327.3333
1399	2013	2017	1,954.00	472	1482	370.5
1400	2013	2017	2,878	816	2062.19	515.5476
1503	2013	2020	10103	5884	4219	602.7143
1566	2013	2019	82,444.00	3,904.10	78539.9	13089.98
1571	2013	2020	3968	227	3741	534.4286
1650	2010	2019	28304	6716	21588	2398.667
1686	2014	2021	3,847.16	1,004.04	2843.118	406.1597
1882	2013	2020	1,017.00	230.00	787	112.4286
1897	2017	2021	33,856.90	8,009.30	25847.6	6461.9
1900	2017	2022	24,641.30	1,111.40	20842	4168.4
2278	2018	2020	NA	NA	7207.22	3603.61

* Reported avoided deforestation is 465 ha larger than the difference between baseline and project deforestation. This is due to differences in the treatment of clouds in satellite imagery during different monitoring periods.

** Reported avoided deforestation is 55 ha larger than the difference between baseline and project deforestation. The reason for this is unclear.

Supplementary table 4. Details of the calculation of avoided deforestation using the numbers produced by PACT for each project included in our assessment. Mean annual deforestation was calculated by summing the total deforestation across the years considered and dividing by the length of the monitoring period, calculated as the difference between the start and end year.

Project ID	Start year	End year	Project Area (ha)	End Year ACC Undisturbed in Project (ha)	End Year ACC Undisturbed in Control Units (ha)	Total Avoided Deforestation (ha)	Mean Annual Avoided Deforestation (ha / yr)
844	2008	2018	101764	91300.75	91027.61	273.1445	27.31445
875	2008	2019	73514.71	71043.43	63877.29	7166.136	651.4669
934	2010	2021	283332.7	225911.9	226927.3	-1015.36	-92.3051
944	2008	2020	179314.7	154773.8	152528.5	2245.265	187.1054
958	2009	2020	300000.2	283137.8	282709.7	428.0313	38.91194
963	2010	2020	36710.17	32268.74	32738.92	-470.181	-47.0181
977	2009	2011	194925.7	190851.7	190757.9	93.80071	46.90035
981	2008	2017	149416.6	136603.4	135564.4	1039.011	115.4457
985	2009	2021	1376941	1328742	1325342	3399.99	283.3325
1047	2007	2012	166280.3	135792.6	130019.1	5773.454	1154.691
1067	2010	2020	592500.1	559263.9	551662.4	7601.468	760.1468
1094	2002	2017	98533.15	94430.82	94592.23	-161.406	-10.7604
1112	2010	2017	43388.84	41956.75	41635.03	321.715	45.95929
1113	2010	2017	29574.87	27484.07	26903.51	580.56	82.93714
1115	2011	2019	77722.75	62981.33	62163.85	817.4765	102.1846
1118	2009	2017	257056	171882.2	183066.9	-11184.7	-1398.09
1122	2009	2012	653764.5	633607.2	633036.3	570.8616	190.2872
1201	2012	2019	71151.99	69093.47	67402.18	1691.295	241.6135
1215	2004	2013	399999.6	353864.1	346472.5	7391.635	821.2927
1311	2007	2011	433047.6	379874.6	371883.8	7990.79	1997.698
1325	2011	2013	68098.36	11.10584	10.21737	0.888467	0.444234
1326	2010	2020	9530.362	7340.2	6791.516	548.6845	54.86845
1329	2011	2019	30138.1	27624.99	23230.32	4394.667	549.3333
1359	2009	2013	187231	179930.6	178387.1	1543.475	385.8688
1360	2010	2019	131818.4	117101.6	120251.1	-3149.42	-349.935
1382	2013	2021	37698.2	36695.86	36834.6	-138.733	-17.3416
1390	2013	2017	142399.7	125050.2	121222.3	3827.924	956.9809

1391	2013	2017	60142.42	56851.88	56796.01	55.86808	13.96702
1392	2013	2017	74893.16	69142.72	68850.33	292.3878	73.09694
1395	2013	2017	129318.8	123321.4	121508.2	1813.248	453.312
1396	2014	2017	85291.4	81134.43	80010.77	1123.659	374.5532
1399	2013	2017	9956.79	9540.3	9443.274	97.02574	24.25644
1400	2013	2017	73303.66	69221.32	68729.69	491.628	122.907
1503	2012	2020	103427.7	89579.41	91936.25	-2356.84	-294.605
1566	2012	2019	1359662	1171476	1164965	6511.406	930.2009
1571	2012	2020	74905.16	71789.92	64239.32	7550.601	943.8252
1650	2009	2019	180410.3	44613.79	32238.9	12374.89	1237.489
1686	2014	2021	190549.5	178282.1	183503	-5221	-745.856

Supplementary table 5. The CO_2e (t) of each ACC land cover class as measured by GEDI in each project. To analyse the effect of different land use transitions within the ACC on carbon and above-ground biomass, we examined several different project areas (n = using the densities pipeline of the PACT v2.1 Method. This entailed taking a 30 km buffer around the project area and sampling for the overlap with the GEDI AGB (tonnes/ha) readings taken in this buffered zone in the year 2020. Filtering for the high-quality readings, we then further reduced our sample to those shots that fell within 3×3 patches tiles of the same land-use class in the ACC raster layer for 2020. With these, we then took the median value for each land use class for each project. We then converted these AGB values to stored CO_2 equivalents (CO_2e) by first multiplying by 1.31 to account for belowground biomass (20%) and leaf litter (11%), then multiplying 0.47 to produce the mean carbon density, and finallying multiplying by 44/12 to produce the mean CO_2e, assuming all stored carbon would be oxidised to CO_2. It is worth noting that some projects were included in this analysis that were absent from the key queries of this study, primarily due to a lack of certified results for them. Project level GEDI derived CO_2e densities are shown in the table below.

Project ID	Undisturbed	Degraded	Deforested	Regrowth
1047	389.28	235.26	65.28	101.87
1094	287.16	45.99	0	52.01
1112	402.51	246.96	0.02	200.93
1113	421.44	298.57	0.07	204.44
1115	597.41	137.8	43.88	21.49
1118	467.74	279.53	0.31	136.86
1122	623.17	188.73	17.04	136.09
1133	463.15	339.21	7.75	244.47
1201	479.62	89.88	45.77	65.75
1215	413	164.42	70.73	178.23
1311	377.77	148.93	52.02	85.42
1326	249.12	93.35	2.4	13.61
1329	262.07	120.99	0	8.96
1359	432.08	78.23	32.93	31.79
1360	403.27	219.63	1.33	103.33
1382	521.41	127.1	0	367.49
1390	341.19	4.43	0	1544.12
1395	344.94	44.91	7.17	96.5
1396	415.36	183.37	6.01	0
1399	512.95	290.86	0	309.9
1503	471.65	116.41	1.37	101.26
1566	308.2	189.42	0.13	98.07
1571	517.06	95.65	2.31	181.92
1650	493.03	316.46	49.72	178.28
1686	542.6	286.51	5.69	378.88
2502	519.54	178.62	1.15	235.27
2558	463.4	136.54	0.18	2.62
3141	478.08	250.89	0	238.6
3347	614.4	429.28	115.98	323.81
674	374.66	50.78	101.17	27
832	438.88	196.42	0.1	77.18
844	507.24	177.48	1.12	213.48
856	599.91	1.22	216.97	84.1
875	453.51	198.33	3.28	42.83
944	383.7	131.2	2.4	33.12
958	498.5	231.18	12.15	29.8

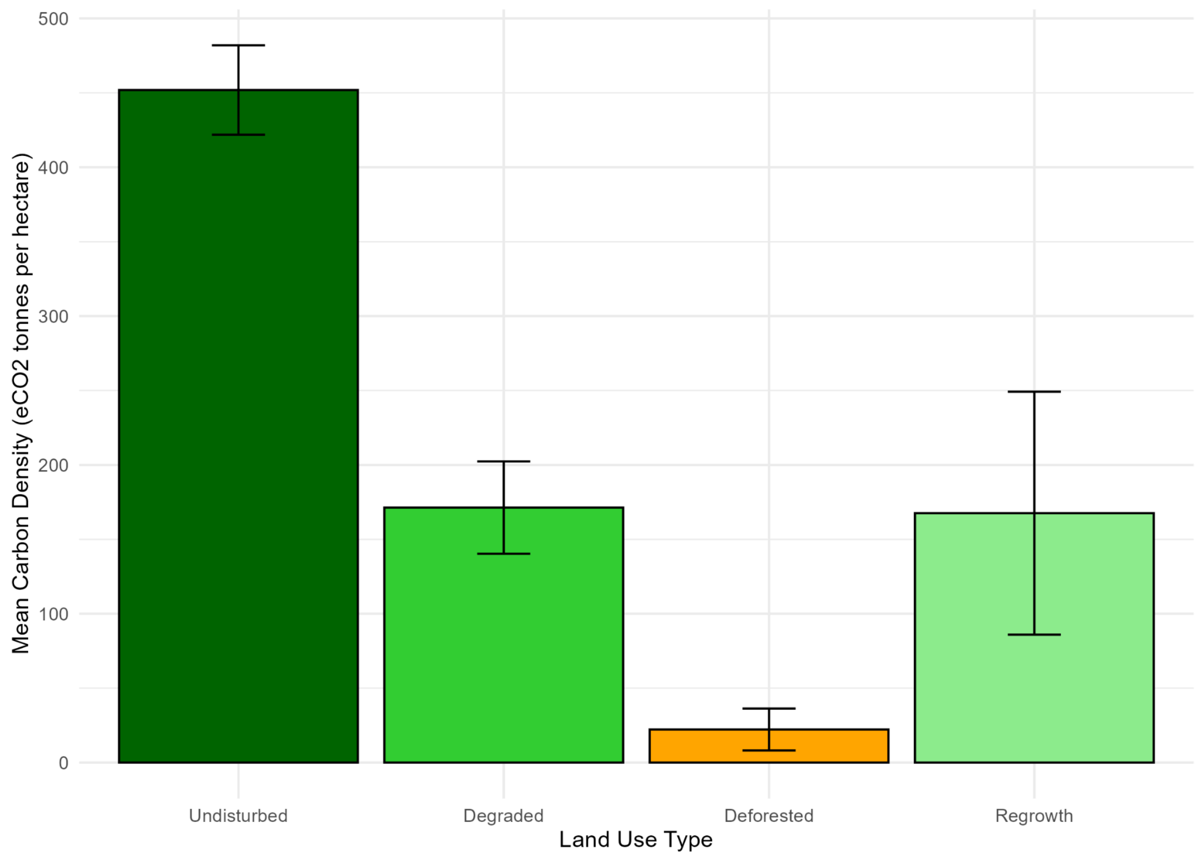

Supplementary figure 24. The mean carbon densities of each ACC forest and cover class as from GEDI across 39 projects. Error bars represent 95% confidence intervals. Both degraded and regrowth classes have a mean CO_2e density < 50% that of the undisturbed class, which was a primary reason to focus on the loss of undisturbed forest in the main analysis.

Supplementary table 6. Details of the calculation of certified compound annual deforestation rates. The amounts of deforestation in ha were taken from figures published in project monitoring documents. The initial forest cover against which rates were derived was determined using the ACC for the start year of the project.

Project ID	Exact Evaluation Period (years)	Project size (ha)	Percent Undisturbed at t0 (%)	Undisturbed Forest in Project at t0	Certified Counterfactual Deforestation Rate (%/year)	Certified Project Deforestation Rate (%/year)
875	10.003	71958.563	99.495	71594.899	2.925	0.309
944	12.005	178294.550	94.645	168746.092	1.177	0.163
958	10.805	296675.526	99.187	294263.524	0.048	0.026
963	9.616	35972.004	96.659	34770.064	1.964	0.552
985	12.405	1357783.248	99.395	1349574.226	0.607	0.006
1094	15.008	98929.399	99.372	98308.162	0.381	0.130
1112	6.797	42695.224	98.662	42123.877	1.326	0.076
1113	6.792	29110.718	96.864	28197.914	2.703	0.184
1115	8.671	78060.622	89.082	69537.589	1.028	0.128
1201	7.419	70222.601	99.668	69989.146	1.861	0.202
1392	4.132	74955.985	93.674	70214.202	1.596	0.308
1395	4.351	129259.792	97.986	126656.771	2.070	0.173
1399	4.518	9843.988	99.872	9831.405	4.786	1.083
1400	4.458	73137.379	96.313	70440.687	0.931	0.261
1566	7.000	1358183.321	89.551	1216270.549	0.998	0.046
1650	10.003	172871.023	40.240	69562.522	5.088	1.010
1047	5.003	144097.414	92.472	133249.242	1.087	0.466
1329	8.005	30175.894	96.006	28970.780	0.970	0.011
1390	3.926	140883.072	93.558	131807.032	0.380	0.091
1396	3.085	84963.637	98.877	84009.427	1.014	0.628
1571	7.603	73438.020	99.742	73248.888	0.730	0.041

Supplementary table 7. Comparison of compound annual deforestation rates in PACT counterfactuals, projects and certified reference areas measured using the ACC. Only 17 projects had certified reference area polygons from which to derive deforestation rates.

Project ID	Compound PACT counterfactual deforestation (%/year)	Compound project ACC deforestation (%/year)	Compound certified reference area ACC deforestation (%/year)
844	0.647	0.558	NA
856	0.332	0.465	NA
875	1.072	0.146	NA
904	5.883	2.384	NA
934	0.843	0.885	NA
944	0.438	0.310	0.740
958	0.158	0.156	0.843
963	0.559	0.712	NA
981	0.466	0.388	NA
985	0.141	0.121	NA
1047	2.005	1.174	3.959
1067	0.243	0.104	NA
1094	0.148	0.146	0.156
1112	0.252	0.140	1.252
1113	0.478	0.202	1.252
1115	0.784	0.637	0.537
1122	0.074	0.048	NA
1201	0.466	0.113	4.436
1215	0.633	0.381	NA
1311	1.426	1.037	NA
1326	2.291	1.677	NA
1329	1.900	0.236	2.643
1359	0.612	0.471	NA
1360	0.685	1.018	NA
1382	0.129	0.170	NA

1390	1.058	0.566	0.574
1391	0.242	0.183	NA
1392	0.147	0.060	0.141
1395	0.444	0.148	NA
1396	0.613	0.245	0.144
1399	0.408	0.209	0.507
1400	0.294	0.130	0.063
1503	1.175	1.280	NA
1566	0.266	0.187	0.762
1571	1.845	0.284	1.110
1650	5.709	2.949	3.691
1686	0.335	0.706	NA
1882	0.405	0.224	NA

Supplementary references

1. Verra. *VCS-Standard 4.4*. https://verra.org/wp-content/uploads/2022/12/VCS-Standard-v4.4-FINAL.pdf (2023).
2. West, T. A. P., Bomfim, B. & Haya, B. K. Methodological issues with deforestation baselines compromise the integrity of carbon offsets from REDD+. *Glob. Environ. Change* **87**, 102863 (2024).
3. Ferraro, P. J. Counterfactual thinking and impact evaluation in environmental policy. *New Dir. Eval.* **2009**, 75–84 (2009).
4. Abadie, A., Diamond, A. & Hainmueller, J. Comparative politics and the synthetic control method. *Am. J. Pol. Sci.* **59**, 495–510 (2015).
5. Schleicher, J. *et al.* Statistical matching for conservation science. *Conserv. Biol.* **34**, (2019).
6. West, T. A. P., Börner, J., Sills, E. O. & Kontoleon, A. Overstated carbon emission reductions from voluntary REDD+ projects in the Brazilian Amazon. *Proc. Natl. Acad. Sci. U. S. A.* **117**, 24188–24194 (2020).
7. Ribas, L. G. S., Pressey, R. L. & Bini, L. M. Estimating counterfactuals for evaluation of ecological and conservation impact: an introduction to matching methods. *Biol. Rev. Camb. Philos. Soc.* **96**, 1186–1204 (2021).
8. Guizar-Coutiño, A., Jones, J. P. G., Balmford, A., Carmenta, R. & Coomes, D. A. A global evaluation of the effectiveness of voluntary REDD+ projects at reducing deforestation and degradation in the moist tropics. *Conserv. Biol.* **36**, e13970 (2022).
9. West, T. A. P. *et al.* Action needed to make carbon offsets from forest conservation work for climate change mitigation. *Science* **381**, 873–877 (2023).
