# Supplementary material for: Learning lessons from over-crediting to ensure additionality in forest carbon credits
Source: Nat Commun. 2026 Apr 11;17:3944. doi: 10.1038/s41467-026-71552-3 (PMC13133336; doi:10.1038/s41467-026-71552-3)
Supplement: Supplementary file 2 — Reporting Summary [file 41467_2026_71552_MOESM2_ESM.pdf]

nature portfolio

Corresponding author(s): Tom Swinfield
Last updated by author(s): 16/02/26

Reporting Summary

Nature Portfolio wishes to improve the reproducibility of the work that we publish. This form provides structure for consistency and transparency in reporting. For further information on Nature Portfolio policies, see our Editorial Policies and the Editorial Policy Checklist.

Statistics

For all statistical analyses, confirm that the following items are present in the figure legend, table legend, main text, or Methods section.

n/a	Confirmed	
☐	☒	The exact sample size (*n*) for each experimental group/condition, given as a discrete number and unit of measurement
☐	☒	A statement on whether measurements were taken from distinct samples or whether the same sample was measured repeatedly
☐	☒	The statistical test(s) used AND whether they are one- or two-sided<br>*Only common tests should be described solely by name; describe more complex techniques in the Methods section.*
☐	☒	A description of all covariates tested
☐	☒	A description of any assumptions or corrections, such as tests of normality and adjustment for multiple comparisons
☐	☒	A full description of the statistical parameters including central tendency (e.g. means) or other basic estimates (e.g. regression coefficient) AND variation (e.g. standard deviation) or associated estimates of uncertainty (e.g. confidence intervals)
☐	☒	For null hypothesis testing, the test statistic (e.g. *F*, *t*, *r*) with confidence intervals, effect sizes, degrees of freedom and *P* value noted<br>*Give P values as exact values whenever suitable.*
☒	☐	For Bayesian analysis, information on the choice of priors and Markov chain Monte Carlo settings
☒	☐	For hierarchical and complex designs, identification of the appropriate level for tests and full reporting of outcomes
☒	☐	Estimates of effect sizes (e.g. Cohen's *d*, Pearson's *r*), indicating how they were calculated

Our web collection on statistics for biologists contains articles on many of the points above.

Software and code

Policy information about availability of computer code

Data collection	Data were compiled from the authors of previous studies and from monitoring reports on the Verra registry.
Data analysis	All analyses were undertaken in R (v4.2.1) using Terra (v1.7.65), Simple Features (v1.0.15) and Raster (v3.6.26) for geospatial processing; Vegan (v2.6.4) for ordination analysis; and the Tidyverse (v2.0.0) for data manipulation.<br>In an effort to contribute to improved transparency, we have made the code necessary to run the PACT evaluations (github.com/quantifyearth/tmf-implementation) and our analysis (github.com/quantifyearth/REDD-Over-Credit-Reasons).

For manuscripts utilizing custom algorithms or software that are central to the research but not yet described in published literature, software must be made available to editors and reviewers. We strongly encourage code deposition in a community repository (e.g. GitHub). See the Nature Portfolio guidelines for submitting code & software for further information.

Data

Policy information about availability of data

All manuscripts must include a data availability statement. This statement should provide the following information, where applicable:

- Accession codes, unique identifiers, or web links for publicly available datasets
- A description of any restrictions on data availability
- For clinical datasets or third party data, please ensure that the statement adheres to our policy

All data necessary to reproduce the analyses in this paper are made publicly available (zenodo.org/records/14895067).

Research involving human participants, their data, or biological material

Policy information about studies with human participants or human data. See also policy information about sex, gender (identity/presentation), and sexual orientation and race, ethnicity and racism.

Reporting on sex and gender	NA
Reporting on race, ethnicity, or other socially relevant groupings	NA
Population characteristics	NA
Recruitment	NA
Ethics oversight	NA

Note that full information on the approval of the study protocol must also be provided in the manuscript.

Field-specific reporting

Please select the one below that is the best fit for your research. If you are not sure, read the appropriate sections before making your selection.

☐ Life sciences ☐ Behavioural & social sciences ☒ Ecological, evolutionary & environmental sciences

For a reference copy of the document with all sections, see nature.com/documents/nr-reporting-summary-flat.pdf

Ecological, evolutionary & environmental sciences study design

All studies must disclose on these points even when the disclosure is negative.

Study description	The study brings together six independent ex-post evaluations of avoided deforestation projects, including a new analysis by the authors to assess the impacts of 44 REDD+ projects. 17 projects had sufficient data for a detailed evaluation of the mechanisms that resulted in different estimates of the amount of avoided deforestation between independent and certified evaluations.
Research sample	The study comprises 44 forest conservation projects, operating in tropical moist forests that had issued carbon credits through the voluntary carbon market.
Sampling strategy	The sample was the largest we could obtain from the REDD+ projects with publicly available geospatial polygons and monitoring reports at the time of evaluation.
Data collection	Data were compiled from the authors of previous studies and from monitoring reports on the Verra registry.
Timing and spatial scale	Data were collected in 2023 and cover project activities spanning 2003-2021. At the time of analysis 2021 was the most recent year for which the remote sensing data we used for measuring deforestation (the European Union's Tropical Moist Forest Annual Change Collection) was available.
Data exclusions	All Verra REDD+ within the tropical moist forest annual change collection coverage were included.
Reproducibility	All code is reproducible and has been reproduced several times successfully.
Randomization	The assessment of avoided deforestation makes use of quasi-experimental methods to control for non-random allocation of areas to REDD+
Blinding	NA

Did the study involve field work? ☐ Yes ☒ No

Reporting for specific materials, systems and methods

We require information from authors about some types of materials, experimental systems and methods used in many studies. Here, indicate whether each material, system or method listed is relevant to your study. If you are not sure if a list item applies to your research, read the appropriate section before selecting a response.

Materials & experimental systems

n/a	Involved in the study
☒	☐ Antibodies
☒	☐ Eukaryotic cell lines
☒	☐ Palaeontology and archaeology
☒	☐ Animals and other organisms
☒	☐ Clinical data
☒	☐ Dual use research of concern
☒	☐ Plants

Methods

n/a	Involved in the study
☒	☐ ChIP-seq
☒	☐ Flow cytometry
☒	☐ MRI-based neuroimaging

Plants

Seed stocks	NA
Novel plant genotypes	NA
Authentication	NA
